# Global Harmonization of Urbanization Measures: Proceed with Care

**DOI:** 10.3390/rs13244973

**Published:** 2021-12-08

**Authors:** Deborah Balk, Stefan Leyk, Mark R. Montgomery, Hasim Engin

**Affiliations:** 1 CUNY Institute for Demographic Research (CIDR), City University of New York, New York, NY 10010, USA; 2 Marxe School of Public and International Affairs, Baruch College, City University of New York, New York, NY 10010, USA; 3 Department of Geography, University of Colorado Boulder, Boulder, CO 80309, USA; 4 Institute of Behavioral Science, University of Colorado Boulder, Boulder, CO 80309, USA; 5 Department of Economics, Stony Brook University, Stony Brook, NY 11794, USA; 6 Population Council, New York, NY 10017, USA; 7 Center for International Earth Science Network (CIESIN), The Earth Institute, Columbia University, Palisades, NY 10964, USA

**Keywords:** demographics, spatial, economic geography, urban economics, spatial demography, urbanness, built environment, rural–urban continuum, remote sensing

## Abstract

By 2050, two-thirds of the world’s population is expected to be living in cities and towns, a marked increase from today’s level of 55 percent. If the general trend is unmistakable, efforts to measure it precisely have been beset with difficulties: the criteria defining urban areas, cities and towns differ from one country to the next and can also change over time for any given country. The past decade has seen great progress toward the long-awaited goal of scientifically comparable urbanization measures, thanks to the combined efforts of multiple disciplines. These efforts have been organized around what is termed the “statistical urbanization” concept, whereby urban areas are defined by population density, contiguity and total population size. Data derived from remote-sensing methods can now supply a variety of spatial proxies for urban areas defined in this way. However, it remains to be understood how such proxies complement, or depart from, meaningful country-specific alternatives. In this paper, we investigate finely resolved population census and satellite-derived data for the United States, Mexico and India, three countries with widely varying conceptions of urban places and long histories of debate and refinement of their national criteria. At the extremes of the urban–rural continuum, we find evidence of generally good agreement between the national and remote sensing-derived measures (albeit with variation by country), but identify significant disagreements in the middle ranges where today’s urban policies are often focused.

## 1. Introduction

The United Nations forecasts that, by 2050, two-thirds of the world’s population will live in cities and towns, well above today’s level of 55 percent, with further increases in store [1]. Rigorous, scientifically justifiable assessments of the changes in urban population and in the extents and forms of the built-up environment will be needed to face the challenges of the coming century, namely, urban and rural economic development [2–6], poverty alleviation and inequality [7–9], disease transmission [10,11], health and health disparities [12,13], carbon emissions [14,15] and climate change [16–20]. These issues demand rigorous investigation across countries and over time—but scientific studies of urban change have often been thwarted by the heterogeneities of national urban definitions [1,21]. Even within the “statistical urbanization” camp, in which urban definitions are couched solely in terms of population density, contiguity and size, there exist divergent views on the proper measurement of urban population and land area [22] and, arguably, an over-reliance on population density as the definitive indicator of urban locations. Recognizing that density measures alone are likely to be inadequate for many purposes, researchers are now beginning to explore the prospects for merging satellite-derived data with on-the-ground socioeconomic measures drawn from spatially disaggregated population censuses and surveys [20,22–26]. A necessary first step in this emerging program of research is to assess areas of agreement and disagreement between global remotely-sensed and country-specific urban concepts and measures.

Although many researchers have lamented the non-comparability of country-specific definitions, there has perhaps been an insufficient appreciation of how these definitions have evolved to reflect national priorities and economic development strategies. Taking a comparative approach, this research study examines three countries that vary significantly in their urban criteria—the United States, Mexico and India. We aim to achieve a better understanding of how the definitions adopted by these countries compare with classifications derived from remotely sensed data [27–31]. Such differences have seldom been systematically quantified and evaluated from the shared perspectives of the scientific communities concerned with local governance, demography, land cover and remote sensing. In particular, given its endorsement in March 2020 by the United Nations Statistical Commission, multiple independent evaluations of the *Degree of Urbanization* method—to be discussed in detail below—are now very much in order [32–34]. This statistical urbanization model has increasing appeal in policy circles, where it is being combined with other types of data to describe the characteristics of population along the urban–rural continuum [24,35,36].

We find that internationally comparable “statistical” portraits of urban areas generally tend to agree with census-based classifications at the extremes of the urban–rural continuum—occupied by large, dense cities on the one end and sparsely-populated rural areas on the other—but with substantial variation by country. There is especially strong agreement in the case of the United States, which has adopted statistical criteria not unlike those now being applied internationally. In all three study countries, the significant challenges lie in the middle of the continuum, where national and internationally comparable systems often disagree. Our analysis underscores the need for new findings on urban land and population to be communicated cautiously, with recognition that the differences between national statistical estimates and those based on remotely sensed data should not be dismissed lightly.

## 2. Materials and Methods

This study compares and combines population-centric and land-structure-centric views of urbanization. At the core of the analysis is the finest-grained spatial unit of analysis for which (*c*. 2010) there exists complete national coverage, namely, Indian settlements (villages, legally constituted towns, census towns and outgrowths) and within-town wards, Mexican basic geographic areas and United States census blocks. Although they apply different definitions in doing so, all three countries assign urban designations to these fine-scaled units, which then become the building-blocks of larger urban entities. The census-based data are complemented by the Global Human Settlement Layer (GHSL-BUILT), a remote sensing-derived product that estimates built-up land area [37]. The land-centric perspective on settlement of GHS-BUILT is then blended with spatial population estimates to produce the *Degree of Urbanization* (DoU) model [32], a well-conceptualized system for urban–rural classification that is potentially applicable across the globe.

### 2.1. The Three Study Countries

Among the three countries we study, the U.S. has perhaps most closely adhered to pure “statistical” criteria in its official urban definitions, whereby a small census unit of land (a census block) is declared urban on the basis of the block’s population density, the identification of contiguous blocks that also meet a density criterion, and the total population of all such blocks. The algorithmic details are well explained by [38], who also recounts the intellectual history leading to these present-day definitions. As is well known—Atlanta, Georgia, is an often-cited case—peri-urban development in the U.S. has been dominated, in many regions, by the suburbanization of middle- and higher-income groups, who possess the means to secure good-quality public services and who connect to core cities and employment by way of automobile-dominant transport networks [39]. A complicating recent development is that poverty also seems to be decentralizing into suburban and urban outlying areas (for a review, see [40]).

Mexico presents a more complicated case. Especially over the 2000–2012 period, national policies and funding strongly encouraged the development of large-scale, low-density housing in urban peripheries, motivated by the desire to improve access to housing for poor and lower-middle-income groups [41]. These well-meaning policies did not cover the costs of land acquisition, an oversight that led to the development of rural plots of land that were only later converted to urban plots on a fragmentary, case-by-case basis. Consequently, many of Mexico’s new low-income communities in the peripheries have lacked essential public services and adequate infrastructure, including transport to more centrally located sites of employment. For much of the time-span of our research study—before the initiation of post-2013 policy reforms—the contrasting situations of the U.S. and Mexico are well summarized by [41] (p. 31):

The resulting sprawl of Mexican cities is different from suburbanization in the United States during the 1960s and 1970s, where middle-class households moved to suburbs for more space with better amenities and schools. Instead, urban growth in Mexico has been connected to the fissure between new, peri-urban developments and more central neighborhoods in terms of the provision of infrastructure and services (including health and education), connectivity, access to sources of employment and urban amenities.

Judged in terms of built-up percentages and population density alone, the patterns of urban sprawl in Mexico and the U.S. might appear to be quantitatively similar, but any such similarities are superficial; the inhabitants of urban peripheries in these two countries differ in their socioeconomic standing, access to public services and connections to employment.

India occupies a distinctive position in the set of study countries. According to the country’s official criteria, its urban percentage in 2011 was only 31.2 percent, well under half the percentages of Mexico and the U.S. at the time. However, in the Indian context, the meaning of “urban” is jurisdictional: urban populations are defined in terms of the people living within the boundaries of legally urban local governments. (A partial exception is made for census towns and outgrowths, which are legally rural despite having urban-like features in terms of density, size and the extent of non-agricultural employment. A legally rural settlement is eligible to be designated as a census town if it has a population expected to be 5000 or above in the upcoming census, an expected density of 400 persons per km^2^ and if 75% of the male main workforce is likely to be engaged in non-agricultural activities. Outgrowths are areas of high-density, arguably urban settlement that are spatially adjacent to statutory cities and towns and which would thus seem to be poised on the threshold of becoming legally urban. In the meantime, however, they too continue to be governed by rural local governments.) Before approving the transition of a rural settlement to legally urban status, Indian state-level authorities must weigh the fiscal costs—loss of access to dedicated, relatively plentiful rural development funds—against the prospects of securing commensurate urban-dedicated funds, which have proven difficult for smaller cities and towns to obtain [42,43].

Perhaps as a consequence, a substantial proportion of India’s rural population lives in large, dense, legally rural villages that elsewhere might be accorded urban status. According to [44–46], in 2011 there were 155,732 Indian villages with at least 1000 population and a density of 400 persons per km^2^ or more, accounting for almost 80 percent of the country’s total rural population. The high densities of these villages raise concerns about the adequacy of sanitation and other services that would normally be addressed through urban infrastructure programs. High rural population densities also present a challenge to density-centered “statistical” definitions of urban.

Some researchers contend that the tight linkage between India’s jurisdictional criteria and its fiscal system causes the country’s urban percentages to be seriously understated [47]. A more sympathetic view is that urban entities in India are regarded as being fundamental units of decentralized governance, a position that was formalized in India’s constitutional amendments of 1993 [43]. Since then, the country has struggled to find an operationally sustainable model for the local end of the urban governance tier.

As this brief account suggests, in the three study countries much recent policy attention has been directed to urban peripheries, smaller urban centers, and larger rural settlements—all of which are situated in the middle ranges of the urban–rural continuum. In these spaces, remotely-sensed methods may well excel in producing rigorous estimates of built-up land and population densities in fine spatial detail. However, for the foreseeable future, such estimates will need to be supplemented with measures of socioeconomic composition, service provision and governance if the densities are to be properly interpreted. In the near term, accepting that remotely-sensed methods will not soon provide persuasive evidence of socioeconomic variation within small spatial units, such measures can at least identify terrain that is rapidly developing over the years between censuses and prompt on-the-ground investigation by governments into the adequacy of services and transport. In this way, the new methods can help alert local authorities to the fast-paced changes underway in their own and nearby jurisdictions.

### 2.2. Official Data Sources

#### 2.2.1. Indian Settlements and Within-Town Wards

Settlement-specific summaries of the population and socioeconomic characteristics of all officially defined Indian settlements covered in its 2011 Census—rural villages, statutory (i.e., legally urban) towns and their wards and the quasi-urban census towns and outgrowths—have been placed in the public domain by the Registrar General of India and are readily accessible via the national census website (https://censusindia.gov.in/2011-Common/CensusData2011.html, accessed on 1 April 2021). Unfortunately, the boundaries of 2011 Indian settlements (and within-town wards) are not yet publicly available. We have relied on a comprehensive collection of settlement boundaries in vector format, originally assembled by the private firm ML Infomap Ltd. (New Delhi, India), whose license allows the data to be displayed in research products but not redistributed (for discussion, see [25]).

#### 2.2.2. Mexican Basic Geographic Areas

Mexico’s official definition of urban begins with the identification of distinct settlements termed *localities*, which are classified as *urban localities* if the settlement contains 2500 or more inhabitants (see the account of [41], pp. 106–107). Each urban locality is spatially subdivided into basic geostatistical areas (AGEBs), which are groups of blocks delimited by streets, avenues, sidewalks, or other easily identifiable construction, in which land is used mainly for occupational, industrial, service provision, or commercial purposes. They are further constrained in size by the requirement that a single census enumerator should be able to canvass an AGEB. The boundaries of AGEBs are typically drawn so as to extend just beyond the current built-up area. Boundary adjustments are then made after each census to keep pace with local land development. All other land area outside urban localities is defined as rural and is spatially divided into rural AGEBs, within which *rural localities* (villages) are situated. To locate urban AGEBs spatially, we have relied on detailed boundary files that the National Institute of Statistics, Geography and Informatics (INEGI) has placed in the public domain (http://en.www.inegi.org.mx/temas/mg/#Downloads, accessed on 1 April 2021).

#### 2.2.3. United States Census Blocks

Not unlike Mexican AGEBs, U.S. census blocks are delineated by both man-made and physical characteristics of the landscape, such as roads and rivers as well as legal and administrative boundaries in some instances. They can differ greatly in area and total population, ranging from zero to several hundred people in cities. As [38] explains, in the 2010 U.S. census an urbanized area (UA) was defined as a contiguous set of blocks each having a population density >1000 people per mi^2^ and, when taken together, a total population in excess of 50,000. Urban clusters (UCs) were defined as a core set of contiguous census blocks with densities greater than 1000 people per mi^2^ but a total population across such blocks of 2500–49,999 persons. Any blocks in close proximity (within 2.5 miles) to UAs and UCs were defined to be urban if their population density exceeded 500 people per mi^2^ (a complex algorithm defines proximity; it allows short “hops” and “skips” to connect otherwise dis-contiguous units). Also defined as urban are some categories of land in industrial and commercial use—non-residential blocks mainly covered by impervious surfaces (pavement, parking lots, and airports) within 0.25 miles of populated urban blocks of UAs and UCs. The boundaries, populations and urban–rural status of all 2010 U.S. census blocks are accessible in the public domain (https://www.census.gov/programs-surveys/geography/technical-documentation/complete-technical-documentation/tiger-geo-line.html, accessed on 1 April 2021).

### 2.3. Data Sources Derived from Remote Sensing

#### 2.3.1. Global Human Settlement Layer

Produced by the Joint Research Centre of the European Commission, GHSL-BUILT estimates built-up land on the basis of Landsat imagery gathered in a series of global snap-shots centered on 1975, 1990, 2000 and 2014. For this research study, we used the 2014 version, whose imagery was assembled some 4 years after the population censuses of Mexico and the U.S. and 3 years after the Indian census. The GHSL team applies machine-learning methods to identify the presence or absence of structures at a resolution of 30 m^2^. Individual structures are not themselves identified, only the proportion of the grid cell occupied by one or more structures. The model has been trained not to mistake roads for structures—it does not currently identify roads as such—and does not (yet) distinguish residential from non-residential structural coverage [30,37,48–50]. Apart from a 30 m^2^ water mask, we used the 250 m^2^ aggregated version of GHSL-BUILT, which provides the proportion of each grid-cell that is built-up. Recent validation efforts have reported acceptable levels of accuracy of GHSL-BUILT except in low-density rural regions [51–54].

#### 2.3.2. Gridded Population of the World and Global Human Settlement Population Layer

Two intermediate products serve to link GHSL-BUILT to the classification system of the *Degree of Urbanization* model. The Gridded Population of the World (GPW) ([55], Version 4.11) is based exclusively on sub-national administrative unit boundaries and total unit populations. To produce the Global Human Settlement Population Grid (GHS-POP), a dasymetric refinement method is applied to reallocate GPW population counts for administrative units to the grid cells within the unit boundaries, according to the finer-resolution, cell-specific proportions built-up as estimated by GHS-BUILT. As [56] note, “The benefit of GHS-POP is that it restricts population to built-up areas and makes its density directly proportional to the density of built-up areas (Freire et al., 2015). However, …, population may be allocated to ‘non-residential’ areas such as commercial, industrial and recreational areas”. The assumption of direct proportionality between population and built-up densities is possibly too strong, although, admittedly, no compelling alternative is yet in hand. Alternative methodological solutions are explored in [57,58]; see [59] for a review of such approaches. Another concern warranting attention is that, due to the limited detection accuracy of GHS-BUILT in thinly settled rural areas, it is possible that GHS-POP over-concentrates administrative-unit population in the more built-up areas of the unit, which is likely to produce overestimates of urban populations relative to rural population when these data are incorporated into the DoU algorithm. Despite this limitation at the rural end of the continuum, recent studies have shown that in urban and urbanized areas, GHS-POP can provide accurate estimates of pixel-level population [52,53,56,60].

#### 2.3.3. The *Degree of Urbanization* Model

The *Degree of Urbanization* (DoU) model further refines GHS-POP by assigning settlement types on the basis of the spatially located population sizes and densities of GHS-POP. Along with other inputs, these data enable the construction of a seven-fold classification of urban and rural settlements [61]. (An eighth category identifies inland open water.) Table 1 presents a simplified version of these seven classes; see [61] for discussion of additional contiguity criteria not spelled out in the table. Note, in particular, the range of settlement types in the band of population density from 300 to 1500 persons per square kilometer; within this band, some difficulties can be anticipated in any effort to cleanly separate *semi-dense urban clusters* and *suburban or peri-urban* settlements from *rural clusters*. Taken together, the seven classes describe the full range of the urban–rural continuum insofar as population density, contiguity and size are concerned.

Additional grid cells qualify for inclusion in the *urban centres* of the DoU model if they are at least 50% built-up. As [61] (p. 18) explain, “This assumption is useful for accommodating the presence in the city of large areas with low resident inhabitants but strongly functionally linked with the city, as for example large productive or commercial areas (typical case of cities in Unite(d) States of America”. A built-up density threshold of 3% was applied to add grid cells to the other three urban categories (*dense urban cluster, semi-dense urban cluster* and *suburban or peri-urban*), with the rationale being given by [61] (p. 19): “Grid cells are included in the urban cluster domain only if some minimal (evidence) of physical built-up structure was recorded by an independent source (with) respect to census data. The purpose of this assumption is to increase the robustness of the GHSL SMOD response by forcing consistency between census-derived sources (population grids) and land cover/land use sources (built-up areas) mitigating the effect of misalignment, thematic bias, scale gaps or other data gaps that may be present in the data”. In short, although the principal density criterion employed in the DoU is population density, a role for built-up density is integrated as well.

The implementation of the DoU depends crucially on the spatial resolution of the GPW administrative unit boundaries containing the unit populations. In the DoU global data set, fine-resolution spatial data are used for the United States and Mexico (census blocks and AGEBs), but only moderate-resolution subdistricts for India (https://sedac.ciesin.columbia.edu/downloads/docs/gpw-v4/gpw-v4-documentation-rev11.pdf, accessed on 1 April 2021). Using restricted-distribution, settlement-level data for India in Supplementary Materials, we illustrate how the resolution of the population data can affect DoU classifications.

#### 2.3.4. Data-Processing

We have conducted all data-processing steps using the Python geoprocessing capabilities of ArcGIS 10.6.1. The comparisons of vector boundary data (corresponding to settlement and within-town ward boundaries in India, the boundaries of AGEBs in Mexico and those of census blocks in the U.S.) to raster data were based on the overlap of vector units with raster cell centroids and employed zonal statistics geoprocessing functions. Vector and raster data for India were projected to *Asia South Albers Equal Area Conic*; for Mexico, the system was *North American Albers Equal Area Conic*; and for the U.S., *USA Contiguous Albers Equal Area Conic*.

### 2.4. Comparing Official Urban–Rural Classes with GHS-BUILT and the DoU

In the final DoU model, the fundamental role of built-up density (that is, the density of structures) is somewhat obscured by the intervening boundary and population layers used in constructing the model. In addition, the dependence of DoU classifications on country-specific administrative boundaries and population counts may limit the application of the DoU method to immediate post-censal periods and to countries that place their boundaries and population counts into the public domain. Hence, there is some value to be gained in a direct assessment of GHSL-BUILT settlement proportions in relation to official urban–rural designations, since the remote-sensing and land classification programs operate independently of census data-collection and, in the future, can be expected to place new estimates in the public domain on a more frequent basis.

To explore an approach based on built-up densities, we have specified a threshold of *τ* = 50 percent built-up to identify potentially urban land (recall that thresholds of 50% and 3% built-up are applied in classifying grid cells in the DoU model, in addition to population densities). Appendix A provides an extensive discussion of threshold choice in what can be viewed as a simple diagnostic test, whereby built-up densities above and below the threshold in a given grid cell serve as an (imperfect) signal of the official urban–rural status of the cell. The entries of Table 2 provide the terms we use in what follows to describe the diagnostic test outcomes in relation to the official urban–rural classification (selected results based on alternative *τ* thresholds are provided in Supplementary Materials).

In extending this approach beyond land classification to address population densities and totals, we have adopted a conventional areal weighting approach [62,63] to distribute population uniformly within the boundaries of each individual census unit of land (settlements or within-city wards in India, AGEBs in Mexico and census blocks in the U.S.). The uniformity assumption may be acceptable within very small census units but has the potential to underestimate population in the highly built-up sections of any given unit and overestimate population in its less built-up sections. However, we do not expect significant errors in the average estimates of population densities, nor do we anticipate systematic biases overall.

#### Official Urban–Rural Classifications and the DoU

**Figure 1.**
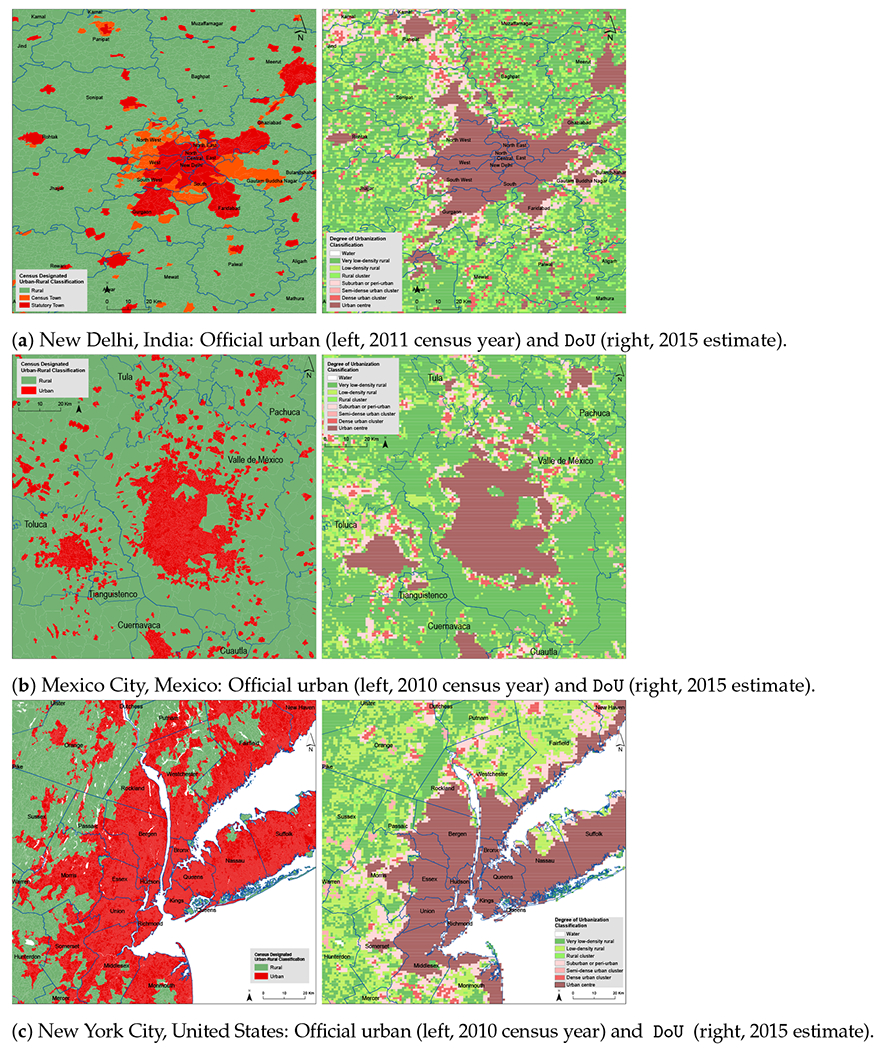
Official urban and *Degree of Urbanization* (DoU) gridded layers. Examples are from (**a**) New Delhi, India; (**b**) Mexico City, Mexico; and (**c**) New York City, United States. All images show a portion of the surrounding area. Administrative boundaries are indicated in blue. These are districts, in the Indian case; municipios, for Mexico; and counties, in the United States.

Similar overlay methods were used to reallocate official population counts to grid cells and thus compare officially classified census units with the more refined seven-fold classification of the DoU. The maps of each of these input layers are shown in Figure 1 for one major city and its surrounding areas in each of the study countries. A visual scan of these examples suggests generally good agreement between the official urban–rural designations and the *Degree of Urbanization* (DoU) classification. However, on closer inspection, areas of nuanced disagreement and ambiguity come into view. For example, in the northern part of Nassau County (to the east of New York City proper), a relatively small patch of land is officially rural, but, in the DoU classification, the rural extents are considerably larger and are intermixed with *suburban or peri-urban* areas. Significant discrepancies are also seen to the south of New York City in Monmouth county (New Jersey), most of which is officially urban yet DoU-classified as rural. The DoU classes (and their descriptive labels) could be regarded as helpful refinements of binary urban–rural official designations, or, when they are not in obvious agreement with such designations, could be taken as an invitation to engage in more detailed critical analysis.

## 3. Results

### 3.1. Classifications of Land

Table 3 summarizes the findings on land area by urban–rural class. Note first that, by official urban criteria, India and the U.S. devote approximately equal percentages of habitable land to urban areas (3.4% and 3.6%, respectively); at 1.2%, Mexico’s allotment is much lower (for international comparisons, see [64]). Of all urban land in India (whether in statutory towns, or in the outgrowths and census towns that, while legally rural, are treated as urban in India’s official tabulations), about 73 percent is occupied by statutory towns, some 3 percent by outgrowths and the remaining 24 percent by census towns (figures not shown). The rural governments that have census towns in their jurisdictions do not have direct access to the urban development funds that could otherwise be used to improve infrastructure and sanitation, nor, in general, can they impose urban rates of property taxation to raise needed revenues.

The *rural agreement* category—using the terminology of Table 2, estimated from a cross-comparison of official census land and GHSL built-up levels—is nearly identical to the figures for officially rural land. This close correspondence means that the sum of the *rural, but built-up, urban, not built-up and urban agreement* categories is also quite close to the officially urban land class. Of all officially urban land area, 58% in Mexico and 41% in the U.S. is found in the *urban agreement* category. However, in India, only 11% of officially urban land is classified as *urban agreement*; the remainder falls into the *urban, not built up* category. In other words, the great majority of officially urban land in India is estimated to be less than half built-up. If this seems surprising and scarcely credible, it may be that our perceptions of high densities in Indian cities are formed mainly by impressions of high *population* densities, rather than by the settlement proportions being described here.

For India, the DoU classes are distributed differently from what can be seen in Mexico and the United States. The most rural category of the DoU, *very low-density rural*, occupies a notably smaller share of Indian habitable land (78.1%) than the 94.8% of land in Mexico and 91.9% in the United States. (The DoU category labels refer mainly to *population* density, not [or at least not directly] to the settlement proportion. In addition, recall that GHSL-BUILT estimates of built-up land in sparsely-settled rural areas—on which the DoU system is based—may well be downwardly biased.) Hence, the remaining rural DoU classes in India account for higher percentages of land than in the other two countries. Another difference worth noting is that, among the three most urban of the DoU land classes (*urban centres, dense urban* and *semi-dense urban*), the *urban centre* class takes a significantly larger shares of land in Mexico (63 percent of these three categories of urban land) and especially the U.S. (72 percent) than in India (only 57 percent).

#### 3.1.1. Built-Up Density by Urban–Rural Category

**Figure 2.**
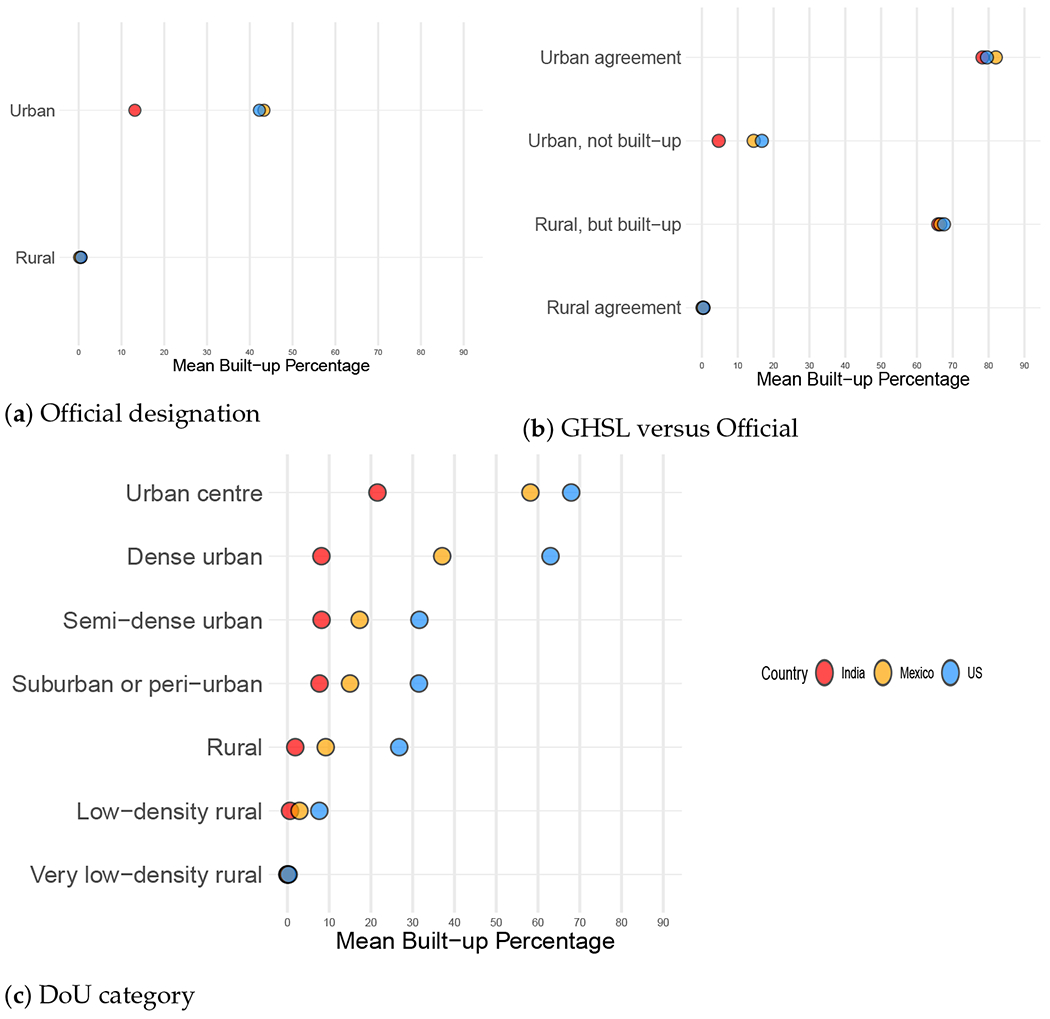
Mean built-up land density by urban–rural category and country. (**a**) Mean built-up densities by official urban–rural designation (2011 census year for India, 2010 for Mexico and the U.S.; 2014 GHSL estimate). (**b**) Comparison of means in the four-cell categorization of GHSL and the official designation. (**c**) Means by DoU category (2014 GHSL and 2015 DoU estimates).

Figure 2 confirms that built-up densities of officially urban land in India fall well below the densities of urban land in Mexico and the United States (panel (a)). As panel (b) shows, the difference is mainly attributable to the low densities of the *urban, not built-up* areas. There is close agreement among the three countries in the mean built-up percentages of the *urban agreement* and *rural agreement* groups and in the *rural, but built-up* categories; only in the *urban, not built-up* category does India appreciably diverge from Mexico and the United States in having lower built-up levels on average. Because we define “built-up” in terms of a *τ* = 50 percent threshold, the averages for *urban agreement* and *rural, but built-up* must exceed 50 percent and the averages for *urban, not built-up* and *rural agreement* must fall short of that threshold. What is striking is the extent to which these categories depart from the threshold value.

Perhaps the most surprising results are those for the built-up densities by DoU class. Figure 2c shows a consistent ranking of the three countries across all DoU classes. The land density percentages in the U.S. are the highest in each class, followed by those of Mexico and India, with a clear pattern of decreasing densities in Mexico and the U.S. as one moves down the urban–rural continuum. However, in India, *suburban or peri-urban, semi-dense urban* and *dense urban* areas exhibit essentially the same settlement proportions, which is noticeably lower than that found in the *urban centres* of the country and of course higher than the three rural DoU classes.

#### 3.1.2. Official Urban Designations by DoU Class

**Figure 3.**
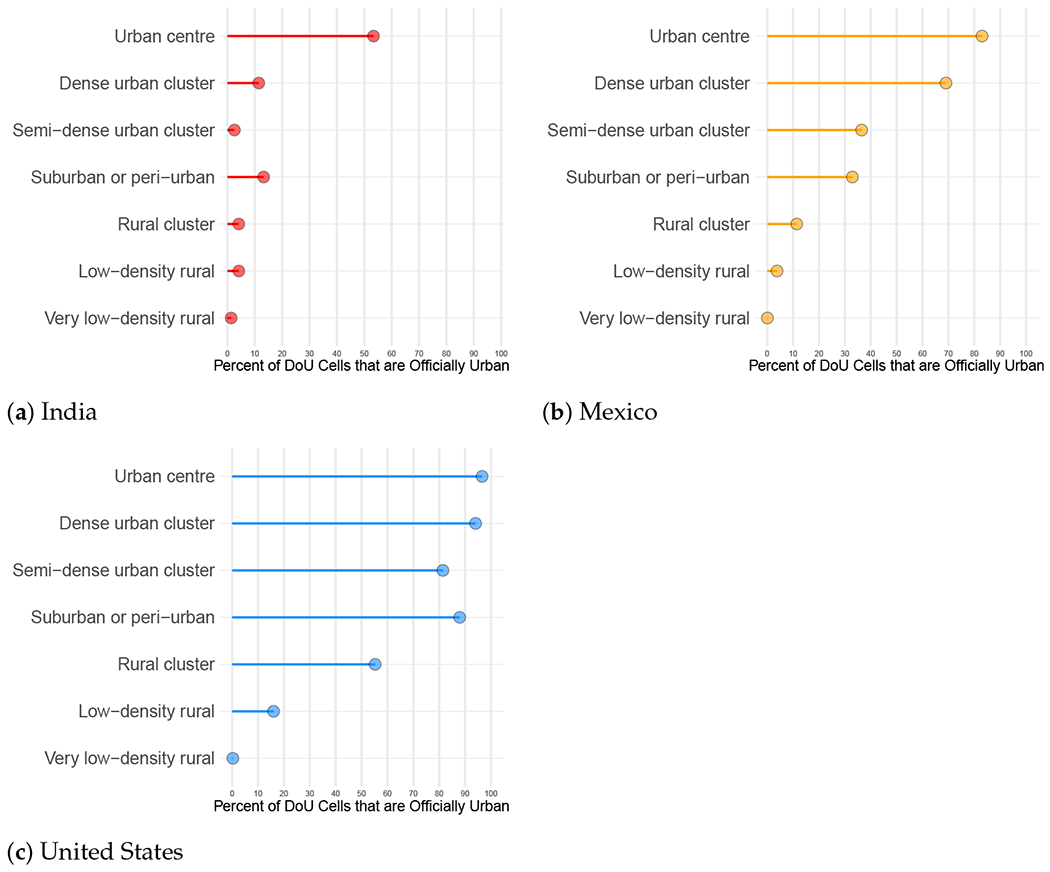
Official urban percentages by DoU class, calculated as the percentage of officially urban cells among all grid cells in each DoU class: (**a**) India, (**b**) Mexico and (**c**) United States. Official figures from 2011 census year for India, 2010 census years for Mexico and the United States. DoU estimate for 2015.

Figure 3 displays one measure of agreement/disagreement between the official urban designations and those derived from the DoU model: the percentages of all grid cells in a given DoU class that are officially urban. At the most rural end of the DoU spectrum, the two classification schemes are in general agreement. However, even in the *rural clusters* class, differences begin to emerge—in the United States, over 55% of grid cells in this DoU class were officially urban—and, moving up the urban–rural continuum in India and Mexico from *suburban or peri-urban* to *urban centres*, a lack of consistent agreement with the DoU becomes evident.

**Figure 4.**
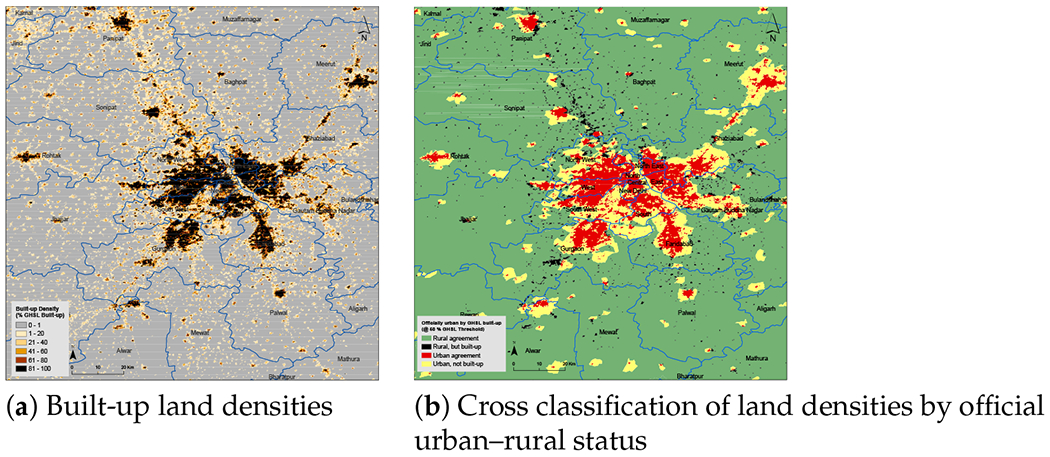
Built-up land densities near New Delhi, India. Official classification for 2011 census year; 2014 GHSL estimate.

**Figure 5.**
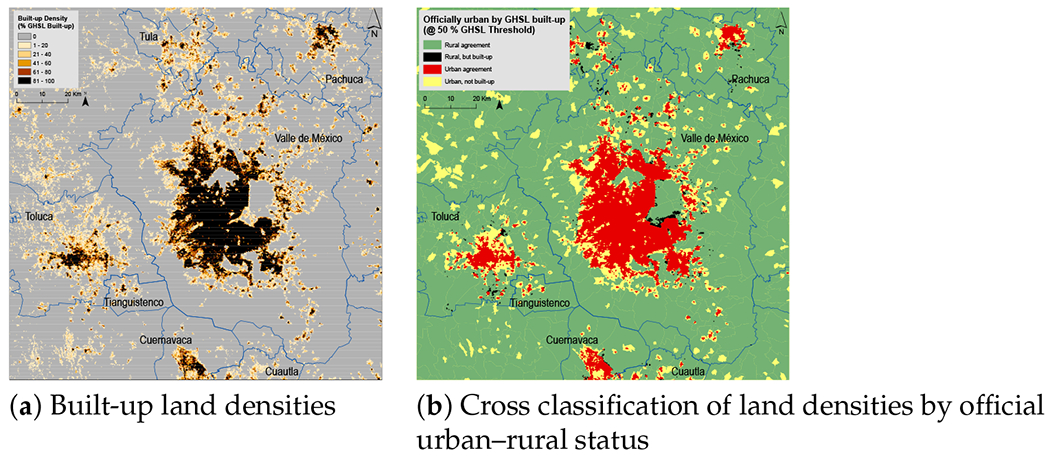
Built-up land densities near Mexico City. Official classification for 2010 census year; 2014 GHSL estimate.

**Figure 6.**
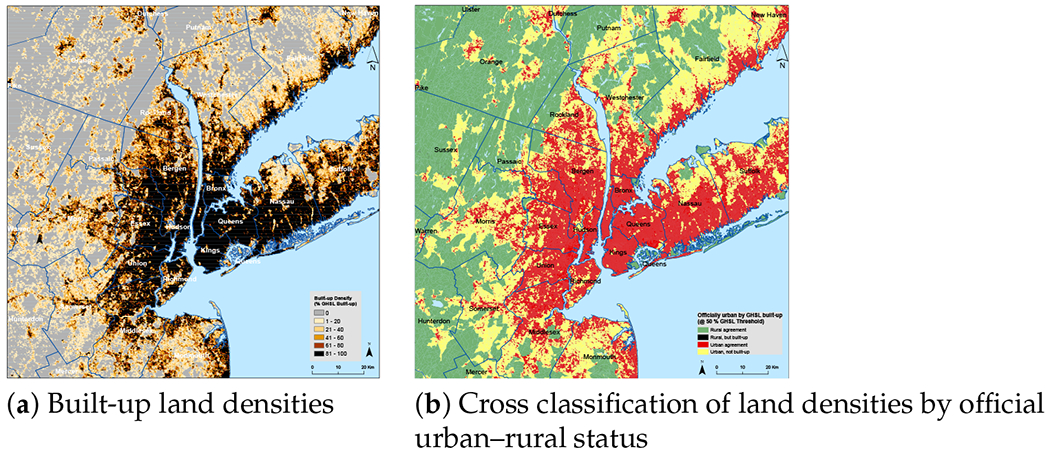
Built-up land densities near New York City. Official classification for 2010 census year; 2014 GHSL estimate.

India presents an array of difficulties for the DoU conception of the urban–rural continuum, not only in defining urban settlements in jurisdictional terms as we have discussed, but also in the percentage of urban-designated land that is less built-up. Figure 4 illustrates the case of New Delhi and its surrounding areas. The areas depicted in yellow and red, when taken together, make up the land officially designated as urban. Evidently, a significant percentage of urban land—even in India’s capital—is less than 50 percent built-up. The composition of urban land near Mexico City—see Figure 5—also includes areas of less built-up land, especially in the smaller urban areas to the west of Mexico City; but as can be seen, Mexico City itself is dominated by built-up land. As with Mexico City, Figure 6 shows that New York City proper (i.e., the five counties that jurisdictionally comprise the city) is nearly all built-up, but, as in New Delhi and to a lesser degree Mexico City, the surrounding areas are officially urban but not majority built-up. In these images (20 km is their common scale), the amount of officially urban land is much larger in the New York metro area—although at a range of built-up land densities—than in the other two regions.

### 3.2. Population Shares and Densities

**Figure 7.**
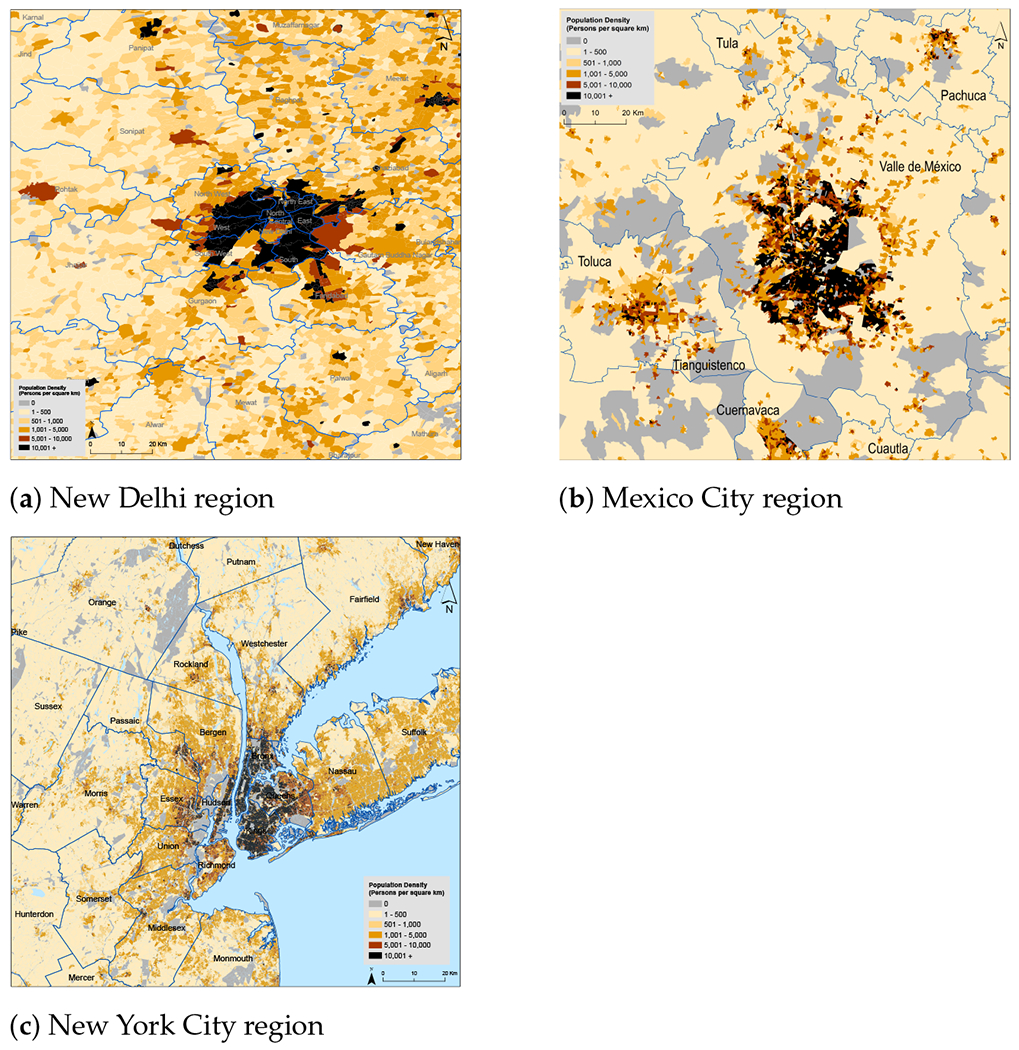
Population densities in selected regions of the study countries. Settlement boundaries and population from the 2011 census year for India (panel (**a**)) and the 2010 census year for both Mexico (panel (**b**)) and the United States (panel (**c**)).

We now turn attention to population and population density, illustrated in Figure 7. This figure shows the spatial distribution of population in New Delhi, Mexico City and New York City and their surrounding areas. For context, the United Nations estimated the 2020 population of the greater urban agglomerations of these cities to be 30.3, 21.8 and 18.8 million, respectively ([1], File 22). The relatively high density of population in India in areas outside the capital, many of which are officially rural (see Figure 1), is clearly evident.

Table 4 displays the total population size and percentage share for each officially designated urban–rural category, the official–GHSL cross-classification and the seven-fold DoU classes. The census-based classification indicate that 77.8% and 80.8% of the population is officially urban in Mexico and the U.S., respectively. As is often remarked-upon, India’s official urban share at the time of its 2011 census, based on legal jurisdictional criteria with additional consideration of census towns and outgrowths, was only 31.3 percent. The official–GHSL cross-classification (built-up threshold 50%), shows that 57–58% of the populations of Mexico and the U.S. lived in places of urban agreement in 2010—areas that were defined as urban by the census and in which built-up density was 50% or greater— whereas in India, only 10.7% of the total population lived in such areas. In all three countries, just over one-fifth of the population (20–24%) lives in areas that were officially urban yet less than 50% built-up. It should be added that, in India, these *urban, not built-up* areas are home to some 248 million people (20.5% of the population), a total that exceeds the number of urban residents of the United States. In the U.S., where 22.8% of people live in the *urban, not built-up* areas, this percentage has been the result of urban expansion (or “sprawl”) into areas occupied by relatively few people but which account for large shares of land [28].

**Figure 8.**
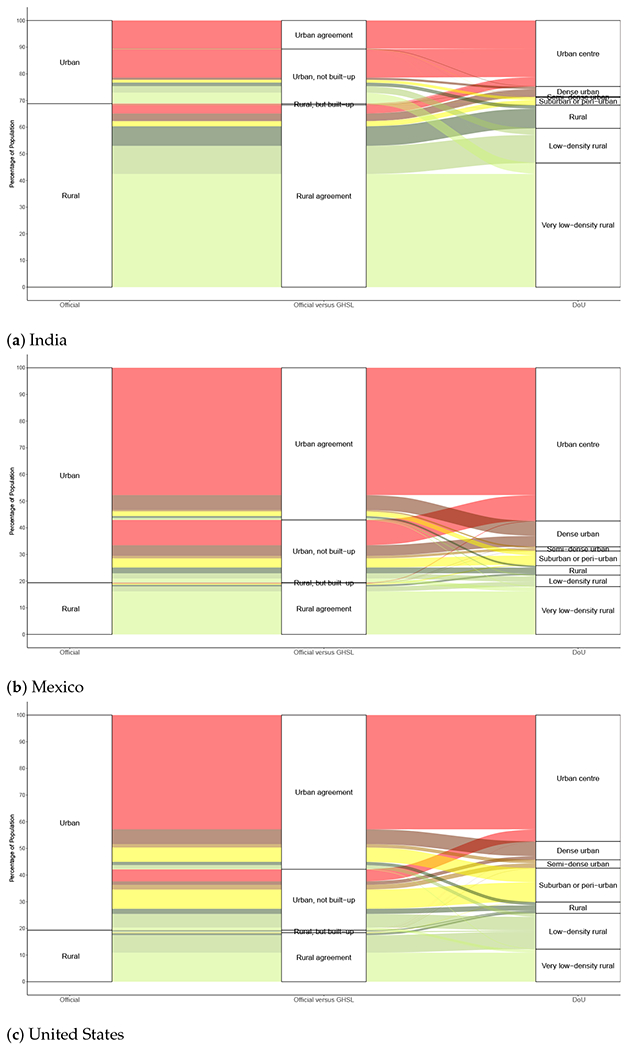
Three-way cross-classifications of official urban–rural categories, GHSL–official cross-classification and DoU classes. Official figures from 2011 census year for India and 2010 census years for Mexico and the United States. GHSL estimate for 2014; DoU estimate for 2015, with classes shown in the order of Table 4.

The more detailed DoU classification place a greater share of India’s population (24.5%) and fewer U.S. residents (47.4%) in *urban centres* than are found in areas of *urban agreement* (a detailed analysis of these differences will be presented in Figure 8 with accompanying discussion). The distribution of the population living in the middle-range urban categories of *dense urban, semi-dense urban* and *suburban or peri-urban* varies considerably across the three countries in ways that do not seem obviously attributable to differences in national urban percentages overall. With only 3.2% of India’s population residing in areas classified as *suburban or peri-urban*, it seems likely that the country’s large, dense villages must be distributed among this class and the *rural* and, possibly, even the *low-density rural* classes.

Table 5 presents the differences in population density by urban classification and country setting. We observe sizable differences across countries in the official urban census-based estimates; India and Mexico have very high urban population densities (>3300 people per km^2^) with much lower densities evident in the officially urban areas of the U.S. (only 886 people per km^2^). As for rural areas, India’s are, respectively, 24- and 33-times denser than the rural areas of Mexico and the United States. In the census–GHSL cross-classification (*τ* = 50% built up), we observe, in areas of *urban agreement*, very high levels of population density in India at 10,283 persons per km^2^ and high levels in Mexico (5336 km^2^), but substantially lower levels in the U.S. (less than 1600 persons per km^2^). Interestingly, population densities in *urban agreement* areas in the U.S. are less than or similar to those found in areas classified as *urban, not built-up* in India (2549) and Mexico (1538), respectively However, the ratios of population densities of these two classes (*urban agreement:urban, not built-up*) are similar in all three countries, ranging between 3.5 and 4.

Highlighting differences between countries, population densities of the *rural, but built-up* areas of India, Mexico and the U.S. are 1101,168 and 57 persons per km^2^, respectively. This suggests two (not mutually exclusive) possibilities: Even small, officially rural settlements may be very densely populated in India; or there may exist many legally rural settlements that might qualify by other criteria to be considered urban. Although the population share of the *rural, but built-up* areas is quite small, there is good reason to think that these built-up rural locations are likely to transition to census-urban over time; see Conclusions for discussion. Areas classified as *rural agreement* (that is, officially rural and below the *τ* = 50 percent threshold) are much less dense in population but still exhibit notable variation across countries—266, 11 and 8 persons per km^2^ in India, Mexico and the U.S., respectively. These results indicate that, while there is general consistency among countries with regard to *ratios* of densities by urban class, the density *levels* of the study countries are quite different.

For the more refined DoU classification (Table 5), we see some curious variations along the urban–rural continuum. Surprisingly, India falls below Mexico in the population densities of *urban centres* and has lower densities than both Mexico and the U.S. in the *dense urban* and *semi-dense urban* categories. This is certainly not expected and may signal some difficulties in applying the DoU approach to urban India. (Recall that the DoU model takes no account of differences in official urban definitions across countries. However, the administrative units used for India are coarser than those employed in the DoU models of the U.S. and Mexico; see the Supplementary Materials.) Further investigation of DoU classification performance is clearly warranted in this case, not least because India accounts for about one-seventh of the world’s population.

### 3.3. A Combined Perspective

In this section, we assess how the DoU classes are composed, by tracing their connections to both the official–GHSL cross-classifications and to the binary urban–rural official designations. The “alluvial plots” of Figure 8 illustrate these three-way linkages, which are expressed in terms of population percentages. The colors of the figure correspond to the seven DoU classes that are shown on the far right of each plot; by following (from right to left) the color flow and the lines (and labels) that demarcate classes, one can trace the composition of these DoU classes (the Supplementary Materials provide the detailed percentages). The colors of the DoU classes are as follows: *urban centres* are shown in red; *dense urban* in brown; *semi-dense urban* in a lighter brown; *suburban or peri-urban* in yellow, marking the divide between urban and rural classes in the DoU scheme; *rural* population is indicated in dark green; *low-density rural* in a lighter green; and *very low-density rural* in the lightest of the green shades (the color scheme suggested in the DoU documentation).

As the figure indicates, *urban centres* and *very low-density rural* areas—the extremes of the urban–rural continuum in the DoU model—tend to agree with the official census-designated layers and census–GHSL cross-classifications. The percentage of the population in the *low-density rural* and *rural* DoU classes in India aligns fairly well with the *rural agreement* class, although a small portion of India’s DoU-*rural, low-density rural* and even *very low-density rural* classes originate in census-designated urban populations of the *urban, not built-up* type. The origins of the DoU *suburban or peri-urban* class in India are one part urban and the other part rural. Curiously, the *semi-dense urban* and *dense urban* DoU classes derive mainly from census-rural populations. However, the *urban centre* class largely originates in census-urban populations, although a significant percentage of this DoU class is traceable back to census-rural populations. In short, there are a number of cross-over cases in India (whereby urban DoU classes stem from census-rural populations, or vice–versa) that call for further analysis. On the whole, such cross-overs are less evident in Mexico, although there certainly exist linkages worth investigating between DoU-*rural* and *low-density rural* populations having origins in census-urban populations. Much the same can be said for the United States, where both DoU-*rural* and *low-density rural* populations can also be traced back to census-urban origins.

Looking across the three countries, it is evident that the *urban, not built-up* class of officially urban populations living on less than 50% built-up land is highly mixed in terms of its representation in multiple urban and rural DoU classes. In setting priorities for further investigation, we would place these *urban, not built-up* areas at the top of the priority list. It is certainly possible that the DoU algorithm successfully refines and correctly labels this important segment of national populations—which, let us recall, accounts for over one-fifth of the total population in each of the study countries—but it is also possible that further tuning of the algorithm will be needed to prevent misclassification.

Likewise, the *urban agreement* cross-classified GHSL–official category is somewhat mixed, contributing to several urban DoU classes and making small contributions to two or more DoU rural classes. Note that, in India, the population of DoU *urban centres* is roughly twice that of the *urban agreement* class; in other words, a substantial share of the *urban centre* population lives on land that is less than half built-up. This can occur in the DoU algorithm for land areas that meet the population size and population density criteria; the 50% built-up test is only applied to grid cells that do not meet these population criteria. A similar if less pronounced pattern is also evident in Mexico.

We should again emphasize that apparent inconsistencies in classification do not in themselves cast doubt on the DoU method. Instead, they may reveal that urban forms are simply too varied to be forced into national, binary, urban–rural containers. Nor is it obvious that apparent mis-classifications in India necessarily contain any lessons for Mexico and the United States—what is considered urban in one country may not be regarded as urban in another. Another complicating factor needs to be mentioned: Rural-like areas can be found amid surrounding urban terrain, such as when natural amenities akin to parks lie within otherwise fully urban areas, a situation that may be expressed in what appears to be misclassification. Finally, as shown in the Supplementary Materials, all these results are dependent on the chosen thresholds of built-up percentages used in both the DoU and the census–GHSL cross-classification and are also dependent on the spatial resolution of the input population data.

## 4. Discussion

In exploring several ways to represent rural–urban classes from population-based, land-based and combined perspectives, we have conducted what is, in effect, one independent evaluation of the DoU classification system, which can be considered the first global data product that represents the full range of the urban–rural continuum. The DoU system is well conceived and, given its purpose, is appropriately free of country-specific urban–rural designations. From one point of view, it can be seen as a diagnostic tool that advances our understanding of official urban differences among countries, with the potential to produce insights that could eventually prompt revision of the official statistics and improve planning and economic development programs [65,66]. Before such benefits can materialize, however, users must first understand precisely how the DoU model departs from country-specific urban conceptualizations. Only then can such a global schema be taken up by countries with differing urban definitions or, indeed, with no urban–rural designations at all.

Our results reveal some similarities among country-specific and harmonized models at the extremes of the urban–rural continuum. In the middle ranges, however, there exist inconsistencies that need to be resolved. Areas that we term *urban, not built-up* have an especially high priority for further investigation. If country differences are most pronounced in this heterogeneous span of the continuum—because, say, growth in population and land-convergence is most likely to occur here, or because these spaces present challenges to transportation or climate adaptation policies—then no global classification model is likely to capture all important local variation. Modern technology, data and methods enable the creation of valuable, scientifically comparable new global datasets—but such datasets must inevitably sacrifice some aspects of local knowledge. Researchers and policy-makers need to be aware of the DoU limitations and constraints to fully understand its fitness for use. The point applies more generally: For all such urban-proxy research programs, both data-producers and the growing community of users stand to benefit from sustained engagement and critical feed-back.

Our research study and findings are subject to several limitations that should be borne in mind. First, it goes almost without saying that the more accurate is the detection of built-up land, the more reliable is the integration of the land perspective in urban definitions. The GHSL-BUILT method is known to face detection and classification challenges in low-density rural areas; improving its performance is the focus of much current research [51,58]. Second, there are concerns about variation in the resolution and units of the population data. As an illustration for India in the Supplementary Materials shows, quite different patterns of DoU classification emerge when the population census data are used at a settlement-level resolution rather than in the coarser subdistrict resolution of the global DoU dataset. Third, as discussed in Appendix A, the appropriate selection of built-up thresholds—which affect not only the GHSL–official cross-classification, but also the composition of the urban classes of the DoU model—needs to be established more rigorously than it has been in the literature. A fourth difficulty has to do with the technique by which population data are allocated spatially; there is potential to further improve the accuracy of the population statistics relative to the dasymetric methods used here. On this point, it has been shown that at least in the global South, the dasymetric refinement of populations using multivariate approaches typically outperforms the simpler GHSL-POP method by integrating additional explanatory variables and implementing different allocation techniques [57,58]. Although the development of advanced methods for population allocation is an active field of research [59,67,68], this on-going research has yet to engage in spatially-explicit studies of place-based characteristics in areas marked by agreement and disagreement between alternative urban classifications. Therefore, it is not yet known whether disagreement is more likely to be found in the peripheries of large or small cities, or in more or less economically developed subnational regions, etc. Focused analyses would help to improve future urban modeling.

## 5. Conclusions

The production of harmonized, globally-comparable statistical measures of urbanization is a remarkable scientific achievement. Even in their first-generation versions, GHSL-BUILT and the *Degree of Urbanization* model exhibit the potential to revolutionize understanding of country-specific urban definitions; night-time lights and similar remotely-sensed measures might add even further value [32,69–75]. In particular, radar-based methods for measuring urban vertical expansion and building volume are likely to complement and extend our understanding of the multiple dimensions of urban growth [76,77]. When they are combined with population and socioeconomic data, such models can produce much-needed, finely-detailed portraits of urbanization along the full urban–rural span. There are excellent near-term prospects for even deeper integration of social and demographic data than the *Degree of Urbanization* model has achieved. In many countries, data to be released in the 2020 round of national population censuses will be more spatially detailed, with more easily accessible boundaries, than was the case at any point in the past. Valuable demographic resources are coming into the public domain; they must be enlisted in the harmonization effort.

While the globally harmonized approaches are being improved, it is vitally important not to overlook the country-specific systems already in place, however difficult they may be to formally integrate. In countries such as India that have adopted jurisdictional criteria to define urban places, legal boundaries delineate the spaces within which local governments possess the authority to act, whether to improve environmental sustainability, safeguard health, or intervene in any number of areas that are high priorities in the Sustainable Development Goals. In the United States, which has fully embraced statistical conceptions of urbanization and expunged jurisdictional names and boundaries from its urban definitions, jurisdictions nevertheless play a central and enduring role in regional policies and governance (see, for example, [78] on core-city populations and their surrounding metropolitan areas). Jurisdictional spaces may never be detectable in remotely-sensed data, but they retain local and national significance. The harmonized measures derived from such data can, however, indicate where urban-like densities of population and structures spill across jurisdictional boundaries to present multiple local and higher-level governments with coordination challenges. As the balance of sustainable development effort shifts from problem description to action and intervention, both jurisdictional and harmonized measures will need to be kept firmly in view.

As our analysis has indicated, much population and land area is located in places that are not obviously either urban or rural. One study of spatial reclassification of urban and rural areas from 1990–2010 found that such areas (in the U.S. case, *rural but built-up* areas) are likely to be reclassified as urban in the next census [79]. We suspect that dynamics such as these will be found to characterize other countries and urban–rural classes. If the pattern seen in the U.S. proves to hold more generally, there may be a land-cover basis for devising spatial forecasts of urbanization for applications ranging from city planning to climate modeling.

In closing, we urge that rural and urban localities be studied not in isolation, but as members of systems, with recognition of and appreciation for their multiple interconnections. A recent and otherwise sophisticated literature on the delineation of specific urban areas, focusing on the use of high-resolution satellite data in high-income, data-rich countries, has surprisingly restricted attention to the identification of individual cities rather than zooming out to survey the wider urban–rural context [80,81]. As the other papers in this Special Issue of *Remote Sensing* demonstrate, the bird’s-eye view provided by remote sensing can contribute not only new measures of urban–rural connections and change, but also new insights with the potential to shape a theory encompassing such disparate units [82–84]. In the lead-up to the adoption of the Sustainable Development Goals, the High-Level Panel [85] argued forcefully for a holistic view of urban–rural–environmental systems and warned of the risks of narrowly urban-specific or rural-specific perspectives:

The post-2015 agenda must be relevant for urban dwellers. Cities are where the battle for sustainable development will be won or lost. Yet the Panel also believes that it is critical to pay attention to rural areas, where three billion near-poor will still be living in 2030. The most pressing issue is not urban versus rural, but how to foster a local, geographic approach to the post-2015 agenda.

To advance toward this long-term goal, the necessary first steps are to achieve an accurate description of the constituent local units and to situate them along an urban–rural continuum—but these are only the first steps.

## Supplementary Material

Harmonization_Supplementary Table S1: Total Area (km^2^) by Degree of Urbanization, using population data of varying spatial resolutions, India, Table S2: Total Population (000’s) by Degree of Urbanization, using population data of varying spatial resolutions, India, Table S3: Total Density (persons/km^2^) by Degree of Urbanization, using population data of varying spatial resolutions, India, Table S4: Total (km^2^) and percentage area by cross-classifications of official urban designations with GHSL, using alternative built-up, Table S5: Total (000s) and percentage population by cross-classifications of official urban designations with GHSL, using alternative built-up thresholds, Table S6: Population density by cross-classifications of official urban designations with GHSL, using alternative built-up thresholds, Table S7: Built-up density by cross-classifications of official urban designations with GHSL, using alternative built-up thresholds, Table S8: Three-way cross-classifications of official urban–rural categories, GHSL–official classification and DoU classes, Mexico (official figures from 2010 census; GHSL estimate for 2014; DoU estimate for 2015), Table S9: Three-way cross-classifications of official urban–rural categories, GHSL–official classification and DoU classes, USA (official figures from 2010 census; GHSL estimate for 2014; DoU estimate for 2015), Table S10:Three-way cross-classifications of official urban–rural categories, GHSL–official classification and DoU classes, India. Upper panel reflects Global DoU based on subdistricts and lower panels reflect DoU using settlement-level administrative data (official figures from 2010 census; GHSL estimate for 2014; DoU estimate for 2015), Figure S1: Degree of urbanization, New Delhi and surrounding areas. Left panel shows DoU distribution using the global data product based on sub-district-level population data; Right panel shows DoU distribution produced with the settlement-level population data, Figure S2: Alluvial Plots India, 25% and 1% thresholds, Figure S3: Alluvial Plots Mexico, 25% and 1% thresholds, Figure S4: Alluvial Plots India, 25% and 1% thresholds, Figure S5: Alluvial Plots India using settlement-level population data, 50%, 25% and 1% thresholds, respectively. References [86,87] are cited in the Supplementary Materials.

## Figures and Tables

**Table 1.** *Degree of Urbanization* (DoU) population density and size criteria. For further detail on continguity criteria, not described here, see [61].

		Minimum Population of the Cluster			No Minimum
		**>50,000**	**5000–50,000**	**500–5000**	
Population density (km^2^)	>1500	Urban centre ①	Dense urban cluster ②	Rural cluster ⑤ ↕	
	300–1500		Semi-dense urban cluster ③		Suburban or peri-urban ④
	50–300				Low density rural ⑥
	<50				Very low density rural ⑦

**Table 2.** Terms used to summarize comparisons between official urban–rural classifications and GHSL built-up percentages, using a built-up threshold of *τ* = 50 percent (see Appendix A).

	GHSL Built-Up Percentage of 250 m^2^ Grid Cell	

Official	≥50%	<50%
Census urban land	*urban agreement*	*urban, not built-up*
Census rural land	*rural, but built-up*	*rural agreement*

**Table 3.** Total and percentage of habitable land area (in square kilometers), by official urban designation, GHSL–official comparisons and DoU category. Official figures from 2011 census year for India and from 2010 census years for Mexico and the United States. GHSL estimate for 2014. DoU estimate for 2015.

	India		Mexico		United States	

Status	Area	Percent	Area	Percent	Area	Percent
	*Official Designation*					

Urban	109,578	3.39	22,942	1.17	274,962	3.58
Rural	3,124,420	96.61	1,942,272	98.83	7,398,680	96.42

	*GHSL threshold versus Official Designation*					

Urban agreement	12,580	0.39	13,237	0.67	113,853	1.46
Urban, not built-up	97,203	3.01	9705	0.49	165,413	2.12
Rural, but built-up	5014	0.16	4872	0.25	13,618	0.17
Rural agreement	3,113,781	96.44	1,937,399	98.58	7,517,111	96.25

	*Degree of Urbanization Category*					

Urban centre	75,094	2.31	12,223	0.63	91,009	1.18
Dense urban	51,522	1.58	4739	0.24	19,496	0.25
Semi-dense urban	4856	0.15	2538	0.13	16,373	0.21
Suburban or peri-urban	41,477	1.27	9010	0.46	64,035	0.83
Rural	234,587	7.21	19,264	0.99	31,426	0.41
Low-density rural	304,857	9.37	52,865	2.72	404,267	5.23
Very low-density rural	2,541,742	78.11	1,844,728	94.83	7,103,698	91.89

**Table 4.** Total and percentage of population by official urban designation, GHSL–official comparisons and DoU category. Total population is expressed in thousands. Official figures from 2011 census year for India and 2010 census years for Mexico and the United States. GHSL estimate for 2014; DoU estimate for 2015.

	India		Mexico		United States	

Status	Population	Percent	Population	Percent	Population	Percent
	*Official Designation*					

Urban	377,108	31.28	85,568	77.82	247,516	80.77
Rural	833,746	68.72	20,685	22.18	59,141	19.23

	*GHSL threshold versus Official Designation*					

Urban agreement	129,362	10.68	60,550	56.99	177,569	57.90
Urban, not built-up	247,747	20.46	25,018	23.55	69,948	22.81
Rural, but built-up	5521	0.46	96	0.09	2287	0.75
Rural agreement	828,225	68.40	20,589	19.38	56,854	18.54

	*Degree of Urbanization Category*					

Urban centre	296,221	24.52	60,861	57.45	144,458	47.37
Dense urban	45,516	3.77	10,235	9.66	21,038	6.90
Semi-dense urban	1988	0.16	1679	1.59	9545	3.13
Suburban or peri-urban	38,501	3.19	5818	5.49	38,801	12.72
Rural	128,965	10.68	3785	3.57	12,905	4.23
Low-density rural	131,087	10.85	4529	4.28	40,953	13.43
Very low-density rural	565,639	46.83	19,026	17.96	37,252	12.22

**Table 5.** Mean population density (persons per square kilometer), by official urban designation, GHSL–official comparisons and DoU category. Official figures from 2011 census year for India and 2010 census years for Mexico and the United States. GHSL estimate for 2014; DoU estimate for 2015.

Status	India	Mexico	United States
	*Official Designation*		

Urban	3388	3730	886
Rural	267	11	8

	*GHSL threshold versus Official Designation*		

Urban agreement	10,283	5336	1560
Urban, not built-up	2549	1538	423
Rural, but built-up	1101	57	168
Rural agreement	266	11	8

	*Degree of Urbanization Category*		

Urban centre	3945	4979	1587
Dense urban	883	2160	1079
Semi-dense urban	409	662	583
Suburban or peri-urban	928	646	606
Rural	549	196	411
Low-density rural	430	86	101
Very low-density rural	223	10	5

**Figure A1.**
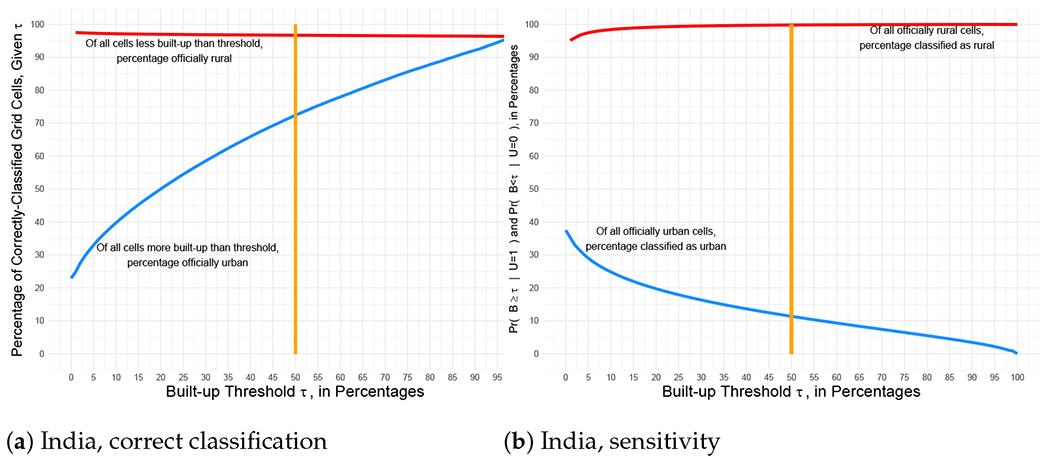
India. Effects of varying *τ* percentage thresholds in the interval (1, 99) on the likelihood of correct urban and rural land classifications and test sensitivity. By “correct”, we mean classifications that are in agreement with the official country-specific urban and rural designations. (**a**) India correct classification percentages by *τ* threshold. (**b**) India test sensitivity percentages by *τ* threshold.

**Figure A2.**
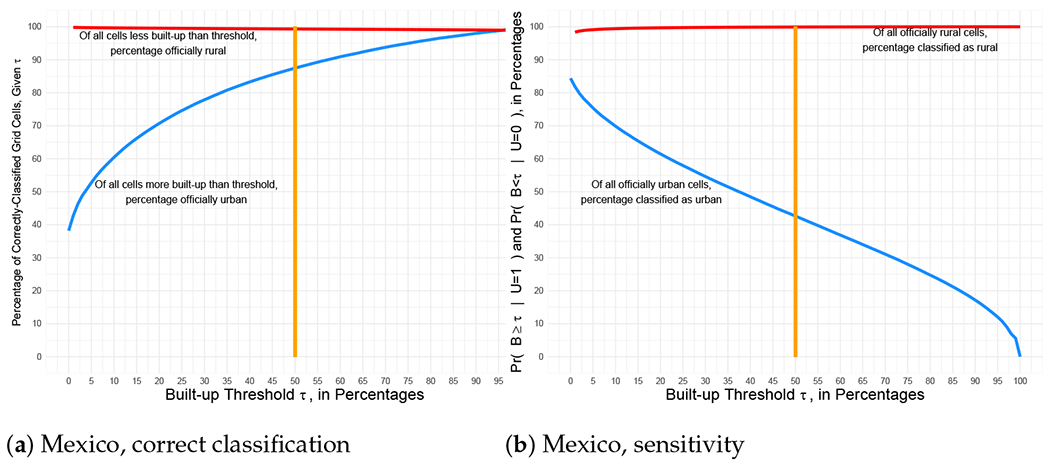
Mexico. Effects of varying *τ* percentage thresholds in the interval (1, 99) on the likelihood of correct urban and rural land classifications and test sensitivity. By “correct”, we mean classifications that are in agreement with the official country-specific urban and rural designations. (**a**) Mexico correct classification percentages by *τ* threshold (**b**) Mexico test sensitivity percentages by *τ* threshold.

## Data Availability

The data used in this study come from the publicly available sources cited throughout.

## Appendix A. A Grid-Cell-Specific Diagnostic Test for Urban and Rural Land

As explained in the main text, the population densities and totals used in the seven-fold DoU model were constructed from the GHSL-BUILT estimates of built-up densities and GPW data on both administrative unit boundaries and total unit populations. The GHSL-BUILT estimates of the density of structures largely determine the spatial placement of GPW population into grid cells within each administrative unit and also determine the level of population density that is assigned to that unit’s built-up cells. To expose the role of built-up density more clearly than is possible in the DoU, we devised a simple thresholding method for grid-cell classification that focuses on the cell’s level of built-up density.

We view the method as a grid-cell-specific diagnostic test based on the percentage of the cell that is built-up. For any selected percentage threshold *τ* ∈ (0, 100), the built-up percentage *B_i_*, in grid cell *i* is compared with *τ*. If *B_i_* ≥ *τ*, the cell is “diagnosed” as being officially urban; if *B_i_* < *τ*, it is diagnosed as officially rural. This test outcome is then checked against the official urban–rural status *U_i_*, of the cell (1 for urban, 0 for rural), using an overlay of urban vector data (polygons) on the GHSL built-up raster.

Table 2 in the main text provides verbal labels for the joint probability distribution of the built-up binary indicator and the official urban binary indicator (for example, the probability of *urban agreement* is Pr((*B_i_* ≥ *τ*) ∩ (*U_i_* = 1))). To go deeper in the exploration of *τ* values, it is helpful to recast the diagnostic problem in terms of conditional probabilities. A good test would have a high conditional probability Pr(*U_i_* = 1|*B_i_* ≥ *τ*) and also a high probability Pr(*U_i_* = 0|*B_i_* < *τ*), both of which must depend on the level of the *τ* threshold that is used. These are the equivalents in the present case of what would be termed “true positives” and “true negatives” in a medical testing context. As the *τ* threshold increases, Pr(*U_i_* = 1|*B_i_* ≥ *τ*) is likely to increase, but Pr(*U_i_* = 0|*B_i_* < *τ*) is apt to decrease. Thus, as *τ* is varied over a range of built-up densities, a generally negative relationship is traced out between the two probabilities.

Two additional probabilities also need consideration, which have to do with the “sensitivities” of the test, i.e., Pr(*B_i_* ≥ *τ*|*U_i_* = 1) (the likelihood that an officially urban grid cell is diagnosed as such by the threshold criterion) and Pr(*B_i_* < *τ*|*U_i_* = 0) (the likelihood that an officially rural cell is diagnosed as rural via the threshold). This pair of probabilities also varies with the *τ* threshold, with urban sensitivity generally declining with *τ* (owing to the frequency of relatively low-density, below-the-threshold urban cells that become excluded from the *B_i_* ≥ *τ* diagnostic) and rural sensitivity increasing.

For these reasons, a test design (i.e., choice of *τ*) that exhibits high values for all four of these probabilities is simply unattainable. How, then, would a single best threshold *τ* be determined? This is not a statistical question as such, but rather a question about decision-making priorities. The optimal *τ* threshold clearly must depend on the positive weights attached by a decision-maker to each of the four relevant probabilities. There is no particular reason to think that decision-makers in different countries, experiencing different urban–rural contexts and paces of change, would necessarily adopt the same weights and arrive at the same choice for the *τ* threshold.

To estimate the four probabilities, we overlay the vector boundaries of officially urban land units on the GHSL-BUILT rasters of built-up density (here, as elsewhere in our analysis, water masks are used to restrict the comparison to habitable units of land). As is readily apparent in Figures A1 and A2, the choice of theshold *τ* has little perceptible influence on the estimated Pr(*U_i_* = 0|*B_i_* < *τ*), the probability of correct rural classification. This is due to two dominating facts about the distribution of land, namely, the percentages of rural land in the national totals for India and Mexico were extremely high—accounting for well above 90 percent of all habitable land—and the built-up percentages of rural land were very low, on average. Urban land misclassified as rural via the *B_i_* < *τ* criterion certainly exists—see the main text for discussion of low built-up densities in India’s officially urban settlements—and the total amount of such misclassified land does increase with *τ*, as would be expected; however, of all grid cells with *B_i_* < *τ* the overwhelming majority remain officially rural even at high values for *τ*. A similar logic explains the lack of a *τ* effect on test sensitivity, Pr(*B_i_* < *τ*|*U_i_* = 0). A large share of rural land has built-up densities below 1 percent, so that raising the threshold above that level has little impact on the percentage of all officially rural land diagnosed as rural by the *B_i_* < *τ* criterion. In short, this kind of threshold test proves to be uninformative where rural land is concerned.

As these figures also show, however, the implications of the *τ* threshold for the correctness and sensitivity of urban land classification are far greater. A tradeoff between “true positives” correctness and sensitivity is clearly apparent; higher *τ* thresholds are associated with higher probabilities of correct classification given density (panel (a) of both figures) but produce lower test sensitivities (panel (b)), because raising *τ* has the effect of excluding relatively low-density urban cells. This presents would-be decision-makers with a dilemma: How should the two urban probabilities be balanced against each other in selecting one *τ* threshold?

Much as with the simple single-threshold diagnostic tool we here describe, issues of correct classification and sensitivity also arise in more heavily parameterized models such as the *Degree of Urbanization*, whose seven categories are based on multiple thresholds for both population density and size, as well as further parameters defining contiguity and built-up density. In this brief treatment, we cannot do justice to the complexities entailed in an analysis of threshold choice in such richer models. These issues must be left to future research.

## References

[1] United Nations, Department of Economic and Social Affairs, Population Division. World Urbanization Prospects: The 2018 Revision; Technical Report; ST/ESA/SER.A/420; United Nations: New York, NY, USA, 2019.

[2] Rakodi C Economic development, urbanization, and poverty. In Urban Livelihoods: A People-Centred Approach to Reducing Poverty; Rakodi C, Lloyd-Jones T, Eds.; Earthscan: London, UK, 2014; Chapter 2.

[3] Pandey B; Seto KC Urbanization and agricultural land loss in India: Comparing satellite estimates with census data. J. Environ. Manag 2015,148, 53–66. 10.1016/j.jenvman.2014.05.014 24958549

[4] Bren d’Amour C; Reitsma F; Baiocchi G; Barthel S; Güneralp B; Erb KH; Haberl H; Creutzig F; Seto KC Future urban land expansion and implications for global croplands. Proc. Natl. Acad. Sci. USA 2017,114, 8939–8944. 28028219 10.1073/pnas.1606036114 PMC5576776

[5] Bakker V; Verburg PH; van Vliet J Trade-offs between prosperity and urban land per capita in major world cities. Geogr. Sustain 2021,2, 134–138.

[6] Forget Y; Shimoni M; Gilbert M; Linard C Mapping 20 Years of Urban Expansion in 45 Urban Areas of Sub-Saharan Africa. Remote Sens. 2021,13, 525.

[7] Mitlin D; Satterthwaite D Urban Poverty in the Global South; Routledge: London, UK, 2013.

[8] Fan P; Ouyang Z; Nguyen DD; Nguyen TTH; Park H; Chen J Urbanization, economic development, environmental and social changes in transitional economies: Vietnam after Doimoi. Landsc. Urban Plan 2019,187,145–155.

[9] Chen M; Sui Y; Liu W; Liu H; Huang Y Urbanization patterns and poverty reduction: A new perspective to explore the countries along the Belt and Road. Habitat. Int 2019, 84,1–14.

[10] Rajashekar A; Bower J Densification without Contagion? Overcrowding and Pandemic Risk Hotspots in Rwanda; Technical Report C19-20082-RWA-1; International Growth Center: London , UK, 2020.

[11] Tian H; Hu S; Cazelles B; Chowell G; Gao L; Laine M; Li Y; Yang H; Li Y; Yang Q; Urbanization prolongs hantavirus epidemics in cities. Proc. Natl. Acad. Sci. USA 2018,115, 4707–4712. 29666240 10.1073/pnas.1712767115 PMC5939059

[12] Montgomery MR Urban Health in Low- and Middle-Income Countries. In Oxford Textbook of Public Health, 5th ed.; Detels R, Beaglehole R, Lansang MA, Gulliford M, Eds.; Oxford University Press: Oxford, UK, 2009; Chapter 10.7, pp. 1376–1394.

[13] Pinchoff J; Mills CW; Balk D Urbanization and health: The effects of the built environment on chronic disease risk factors among women in Tanzania. PLoS ONE 2020, 15, e0241810. 33141863 10.1371/journal.pone.0241810 PMC7608895

[14] Moran D; Kanemoto K; Jiborn M; Wood R; Többen J; Seto KC Carbon footprints of 13,000 cities. Environ. Res. Lett 2018, 13, 064041.

[15] Creutzig F; Baiocchi G; Bierkandt R; Pichler PP; Seto KC Global typology of urban energy use and potentials for an urbanization mitigation wedge. Proc. Natl. Acad. Sci. USA 2015, 112, 6283–6288. 25583508 10.1073/pnas.1315545112 PMC4443311

[16] Stewart ID; Oke TR Local Climate Zones for Urban Temperature Studies. Bull. Am. Meteorol. Soc 2012, 93, 1879–1900.

[17] Revi A; Satterthwaite DE; Aragón-Durand F; Corfee-Morlot J; Kiunsi RB; Pelling M; Roberts DC; Solecki W Urban Areas. In Climate Change 2014: Impacts, Adaptation, and Vulnerability. Part A: Global and Sectoral Aspects. Contribution of Working Group II to the Fifth Assessment Report of the Intergovernmental Panel on Climate Change; Field C, Barros V, Dokken D, Mach K, Mastrandrea M, Bilir T, Chatterjee M, Ebi K, Estrada Y, Genova R, , Eds.; Cambridge University Press: Cambridge, UK; New York, NY, USA, 2014; pp. 535–612.

[18] Solecki W; Seto KC; Balk D; Bigio A; Boone CG; Creutzig F; Fragkias M; Lwasa S; Marcotullio P; Romero-Lankao P; A conceptual framework for an urban areas typology to integrate climate change mitigation and adaptation. Urban Clim. 2015, 14, 116–137.

[19] Demuzere M; Bechtel B; Middel A; Mills G Mapping Europe into local climate zones. PLoS ONE 2019, 14, e0214474. 31017939 10.1371/journal.pone.0214474 PMC6481911

[20] Gao J; O’Neill BC Mapping global urban land for the 21st century with data-driven simulations and Shared Socioeconomic Pathways. Nat. Commun 2020, 11, 2302. 32385275 10.1038/s41467-020-15788-7 PMC7210308

[21] Buettner T Urban Estimates and Projections at the United Nations: The Strengths, Weaknesses, and Underpinnings of the World Urbanization Prospects. Spat. Demogr 2015, 3, 91–108.

[22] Uchiyama Y; Mori K Methods for specifying spatial boundaries of cities in the world: The impacts of delineation methods on city sustainability indices. Sci. Total Environ 2017, 592, 345–356. 28319721 10.1016/j.scitotenv.2017.03.014

[23] Ehrlich D; Freire S; Melchiorri M; Kemper T Open and Consistent Geospatial Data on Population Density, Built-Up and Settlements to Analyse Human Presence, Societal Impact and Sustainability: A Review of GHSL Applications. Sustainability 2021, 13, 7851.

[24] Henderson JV; Liu V; Peng C; Storeyguard A Demographic and Health Outcomes by Degree of Urbanisation: Perspectives from a New Classification of Urban Areas; Paper Prepared for European Commission, Directorate-General for Regional and Urban Policy; London School of Economics and Public Policy: London, UK, 2019.

[25] Balk D; Montgomery MR; Engin H; Lin N; Major E; Jones B Urbanization in India: Population and Urban Classification Grids for 2011. Data 2019, 4, 35. 37424897 10.3390/data4010035 PMC10327898

[26] Leyk S; Uhl JH; Connor DS; Braswell AE; Mietkiewicz N; Balch JK; Gutmann M Two centuries of settlement and urban development in the United States. Sci. Adv 2020, 6, aba2937. 10.1126/sciadv.aba2937 PMC7269677 32537503

[27] Balk D; Leyk S; Jones B; Montgomery MR; Clark A Understanding Urbanization: A Study of Census and Satellite-derived Urban Classes in the United States, 1990–2010. PLoS ONE 2018, 13, e0208487. 30586443 10.1371/journal.pone.0208487 PMC6306171

[28] Leyk S; Balk D; Jones B; Montgomery MR; Engin H The heterogeneity and change in the urban structure of metropolitan areas in the United States, 1990–2010. Sci. Data 2019, 6, 321. 31844062 10.1038/s41597-019-0329-6 PMC6915769

[29] Dijkstra L; Poelman H A Harmonised Definition of Cities and Rural Areas: The New Degree of Urbanisation; Regional Working Paper, Directorate-General for Regional and Urban Policy; European Commission: Brussels, Belgium, 2014.

[30] Freire S; Kemper T; Pesaresi M; Florczyk AJ; Syrris V Combining GHSL and GPW to Improve Global Population Mapping; Conference Paper; EC JRC Global Security and Crisis Management Unit, Joint Research Commission: Ispra, Italy, 2015.

[31] Maffenini L; Schiavina M; Melchiorri M; Pesaresi M; Kemper T GHS-DUG User Guide; Technical Report; Joint Research Commission, Publications Office of the European Union: Luxembourg, 2020.

[32] Dijkstra L; Florczyk AJ; Freire S; Kemper T; Melchiorri M; Pesaresi M; Schiavina M Applying the Degree of Urbanisation to the globe: A new harmonised definition reveals a different picture of global urbanisation. J. Urban Econ 2020, 125, 103312.

[33] United Nations Statistical Commission. Report on the Fifty-First Session (3–6 March 2020); Supplement No. 4, E/2020/24-E/CN.3/2020/37; Economic and Social Council Official Records; United Nations Statistical Commission: New York, NY, USA, 2020.

[34] Statham T; Fox S; Wolf LJ Identifying urban areas: A new approach and comparison of national urban metrics with gridded population data. Comput. Environ. Urban Syst 2021,

[35] OECD; European Commission. Cities in the World; OECD and European Commission: Brussels, Belgium, 2020; p. 160.

[36] Coalition for Urban Transitions. Climate Emergency/Urban Opportunity; Coalition for Urban Transitions c/o World Resources Institute: Washington, DC, USA, 2019.

[37] Corbane C; Pesaresi M; Kemper T; Politis P; Florczyk AJ; Syrris V; Melchiorri M; Sabo F; Soille P Automated Global Delineation of Human Settlements from 40 Years of Landsat Satellite Data Archives. Big Earth Data 2019, 3, 140–169.

[38] Ratcliffe M A Century of Delineating a Changing Landscape: The Census Bureau’s Urban and Rural Classification, 1910 to 2010. Presented at the Annual Meeting of the Social Science History Association, Baltimore, MD, USA, 14 November 2015.

[39] Redding SJ Suburbanization in the United States 1970–2010; Working Paper 28841; National Bureau of Economic Research (NBER): Cambridge, MA, USA, 2021.

[40] Allard SW; Paisner SC The Rise of Suburban Poverty. In Oxford Handbooks Online; Oxford University Press: Oxford, UK, 2016.

[41] Kim Y; Zangerling B (Eds.) Mexico Urbanization Review: Managing Spatial Growth for Productive and Livable Cities in Mexico; Directions in Development; World Bank: Washington, DC, USA, 2016.

[42] Khan S The Other Jawaharlal Nehru National Urban Renewal Mission: What Does It Mean for Small Town India? In Subaltern Urbanisation In India: An Introduction to the Dynamics of Ordinary Towns; Denis E, Zérah MH, Eds.; Springer: New Delhi, India, 2017; Chapter 13, pp. 337–370.

[43] Mathur OP; Naqvi AH; Laroiya A; Sayukta VS; Verma H State of the Cities: India; Institute of Social Sciences: New Delhi, India, 2021.

[44] Dasgupta S; Roy SN; Bhol A; Raj D Towards a New Research and Policy Paradigm: An Analysis of the Sanitation Situation in Large Dense Villages; CPR Research Report; Centre for Policy Research: New Delhi, India, 2017.

[45] Bhol A; Dasguta S; Mukherjee A; Jain A Sanitation in Large and Dense Villages of India: The Last Mile and Beyond; Technical Report, CPR White Paper; Centre for Policy Research: New Delhi, India, 2019.

[46] Denis E; Zérah MH (Eds.) Subaltern Urbanisation in India: An Introduction to the Dynamics of Ordinary Towns; Exploring Urban Change in South Asia; Springer: New Delhi, India, 2017.

[47] Onda K; Sinha P; Gaughan AE; Stevens FR; Kaza N Missing millions: Undercounting urbanization in India. Popul. Environ 2019, 41, 126–150. 31929670 10.1007/s11111-019-00329-2 PMC6934249

[48] Pesaresi M; Huadong G; Blaes X; Ehrlich D; Ferri S; Gueguen L; Halkia M; Kauffmann M; Kemper T; Lu L; A Global Human Settlement Layer from Optical HR/VHR RS Data: Concept and First Results. IEEE J. Sel. Top. Appl. Earth Obs. Remote Sens 2013, 6, 2102–2131.

[49] Pesaresi M; Syrris V; Julea A A New Method for Earth Observation Data Analytics based on Symbolic Machine Learning. Remote Sens. 2016, 8, 399.

[50] Corbane C; Florczyk A; Pesaresi M; Politis P; Syrris V GHS-BUILT R2018A—GHS Built-Up Grid, Derived from Landsat, Multitemporal (1975–1990–2000–2014); European Commission, Joint Research Centre (JRC): Ispra, Italy, 2018.

[51] Leyk S; Uhl JH; Balk D; Jones B Assessing the accuracy of multi-temporal built-up land layers across rural-urban trajectories in the United States. Remote Sens. Environ 2018, 204, 898–917. 29599568 10.1016/j.rse.2017.08.035 PMC5868966

[52] Liu C; Huang X; Zhu Z; Chen H; Tang X; Gong J Automatic extraction of built-up area from ZY3 multi-view satellite imagery: Analysis of 45 global cities. Remote Sens. Environ 2019, 226, 51–73.

[53] Milesi C; Churkina G Measuring and Monitoring Urban Impacts on Climate Change from Space. Remote Sens. 2020,12, 3494.

[54] Uhl JH; Connor DS; Leyk S; Braswell AE A century of decoupling size and structure of urban spaces in the United States. Communications Earth & Environment 2021, 2, 20. 34970647 10.1038/s43247-020-00082-7 PMC8716013

[55] CIESIN. Gridded Population of the World, Version 4 (GPWv4): Population Density, Revision 11; Center for International Earth Science Information Network (CIESIN), Columbia University: Palisades, NY, USA, 2018.

[56] Archila Bustos MF; Hall O; Niedomysl T; Ernstson U A pixel level evaluation of five multitemporal global gridded population datasets: A case study in Sweden, 1990–2015. Popul. Environ 2020, 42, 255–277.

[57] Reed FJ; Gaughan AE; Stevens FR; Yetman G; Sorichetta A; Tatem AJ Gridded Population Maps Informed by Different Built Settlement Products. Data 2018, 3, 33. 33344538 10.3390/data3030033 PMC7680951

[58] Stevens FR; Gaughan AE; Nieves JJ; King A; Sorichetta A; Linard C; Tatem AJ Comparisons of two global built area land cover datasets in methods to disaggregate human population in eleven countries from the global South. Int. J. Digit. Earth 2020, 13, 78–100.

[59] Leyk S; Gaughan AE; Adamo SB; de Sherbinin A; Balk D; Freire S; Rose A; Stevens FR; Blankespoor B; Frye C; The spatial allocation of population: A review of large-scale gridded population data products and their fitness for use. Earth Syst. Sci. Data 2019, 11, 1385–1409.

[60] Calka B; Bielecka E GHS-POP Accuracy Assessment: Poland and Portugal Case Study. Remote Sens. 2020, 12, 1105.

[61] Florczyk A; Corbane C; Ehrlich D; Freire S; Kemper T; Maffenini L; Melchiorri M; Pesaresi M; Politis P; Schiavina M; GHSL Data Package 2019; Technical Report, EUR 29788EN; Publications Office of the European Union: Luxembourg, 2019.

[62] Markoff J; Shapiro G The Linkage of Data Describing Overlapping Geographical Units. Hist. Methods Newsl 1973, 7, 34–46.

[63] Goodchild MF; Lam NSN Areal Interpolation: A Variant of the Traditional Spatial Problem. Geo-Processing 1980, 1, 297–312.

[64] Liu Z; He C; Zhou Y; Wu J How much of the world’s land has been urbanized, really? A hierarchical framework for avoiding confusion. Landsc. Ecol 2014, 29, 763–771.

[65] Mudau N; Mwaniki D; Tsoeleng L; Mashalane M; Beguy D; Ndugwa R Assessment of SDG Indicator 11.3.1 and Urban Growth Trends of Major and Small Cities in South Africa. Sustainability 2020, 12, 7063.

[66] Schiavina M; Melchiorri M; Corbane C; Florczyk AJ; Freire S; Pesaresi M; Kemper T Multi-Scale Estimation of Land Use Efficiency (SDG 11.3.1) across 25 Years Using Global Open and Free Data. Sustainability 2019, 11, 5674.

[67] Mennis J Dasymetric Mapping for Estimating Population in Small Areas. Geogr. Compass 2009, 3, 727–745.

[68] Zandbergen PA; Ignizio DA Comparison of Dasymetric Mapping Techniques for Small-Area Population Estimates. Cartogr. Geogr. Inf. Sci 2010, 37, 199–214.

[69] Min B Power and the Vote: Elections and Electricity in the Developing World; Cambridge University Press: Cambridge, UK, 2015.

[70] Wang P; Huang C; Brown de Colstoun EC Mapping 2000–2010 Impervious Surface Change in India Using Global Land Survey Landsat Data. Remote Sens. 2017, 9, 366.

[71] Koren O; Sarbahi AK State Capacity, Insurgency, and Civil War: A Disaggregated Analysis. Int. Stud. Q 2018, 62, 274–288.

[72] Hu Y; Yao J Illuminating Economic Growth; Technical Report, International Monetary Fund, Working Paper No. 19/77; IMF: Washington, DC, USA, 2019.

[73] Small C International Earth Science Information Network (CIESIN). VIIRS Plus DMSP Change in Lights; Columbia University, Center for International Earth Sciences Information Network (CIESIN): Palisades, NY, USA, 2020.

[74] Ch R; Martin DA; Vargas JF Measuring the size and growth of cities using nighttime light. J. Urban Econ 2020, 125, 103254.

[75] Li X; Zhou Y; Zhao M; Zhao X A harmonized global nighttime light dataset 1992–2018. Sci. Data 2020, 7, 168. 32499523 10.1038/s41597-020-0510-y PMC7272434

[76] Mahtta R; Mahendra A; Seto KC Building up or spreading out? Typologies of urban growth across 478 cities of 1 million+. Environ. Res. Lett 2019, 14, 124077.

[77] Balk DL; Nghiem SV; Jones BR; Liu Z; Dunn G Up and out: A multifaceted approach to characterizing urbanization in Greater Saigon, 2000–2009. Landsc. Urban Plan 2019, 187, 199–209.

[78] Rowlands DW; Loh TH Reinvesting in Urban Cores Can Revitalize Entire Regions; Metropolitan Policy Program Report; The Brookings Institution: Washington, DC, USA, 2021.

[79] Jones B; Balk D; Leyk S Urban Change in the United States, 1990–2010: A Spatial Assessment of Administrative Reclassification. Sustainability 2020, 12, 1649.

[80] Arribas-Bel D; Garcia-López M; Viladecans-Marsal E Building(s and) cities: Delineating urban areas with a machine learning algorithm. J. Urban Econ 2019, 125, 103217.

[81] de Bellefon MP; Combes PP; Duranton G; Gobillon L; Gorin C Delineating urban areas using building density. J. Urban Econ 2019, 125, 103226.

[82] Melchiorri M; Florczyk AJ; Freire S; Schiavina M; Pesaresi M; Kemper T Unveiling 25 Years of Planetary Urbanization with Remote Sensing: Perspectives from the Global Human Settlement Layer. Remote Sens. 2018, 10, 768.

[83] Yang F; Wang Z; Yang X; Liu Y; Liu B; Wang J; Kang J Using Multi-Sensor Satellite Images and Auxiliary Data in Updating and Assessing the Accuracies of Urban Land Products in Different Landscape Patterns. Remote Sens. 2019, 11, 2664.

[84] Brenner N; Schmid C The ‘Urban Age’ in Question. Int. J. Urban Reg. Res 2014, 38, 731–755.

[85] United Nations. A New Global Partnership: Eradicate Poverty and Transform Economics through Sustainable Development. The Report of the High-Level Panel of Eminent Persons on the Post-2015 Development Agenda; United Nations: New York, NY, USA, 2013.

[86] Balk D; Montgomery MR; McGranahan G; Todd M Understanding the Impacts of Climate Change: Linking Satellite and Other Spatial Data with Population Data. In Population Dynamics and Climate Change; Guzman JM, Martine G, McGranahan G, Shensul D, Tacoli C, Eds.; United Nations Fund for Population Activities (UNFPA) and International Institute for Environment and Development (IIED): New York, NY, USA, 2009; Chapter 13, pp. 206–217.

[87] Tatem AJ; Campiz N; Gething PW; Snow RW; Linard C The effects of spatial population dataset choice on estimates of population at risk of disease. Popul. Health Metrics 2011, 9, 4. 10.1186/1478-7954-9-4 PMC3045911 21299885

