# Supplementary material for: Global Harmonization of Urbanization Measures: Proceed with Care
Source: Remote Sens (Basel). Author manuscript; Available in PMC 2023 Jul 7. (PMC10328085; doi:10.3390/rs13244973)
Supplement: Harmonization_Supplementary — Table S1: Total Area (km2) by Degree of Urbanization, using population data of varying spatial resolutions, India, Table S2: Total Population (000’s) by Degree of Urbanization, using population data of varying spatial resolutions, India, Table S3: Total Density (persons/km2) by Degree of Urbanization, using population data of varying spatial resolutions, India, Table S4: Total (km2) and percentage area by cross-classifications of official urban designations with GHSL, using alternative built-up, Table S5: Total (000s) and percentage population by cross-classifications of official urban designations with GHSL, using alternative built-up thresholds, Table S6: Population density by cross-classifications of official urban designations with GHSL, using alternative built-up thresholds, Table S7: Built-up density by cross-classifications of official urban designations with GHSL, using alternative built-up thresholds, Table S8: Three-way cross-classifications of official urban–rural categories, GHSL–official classification and DoU classes, Mexico (official figures from 2010 census; GHSL estimate for 2014; DoU estimate for 2015), Table S9: Three-way cross-classifications of official urban–rural categories, GHSL–official classification and DoU classes, USA (official figures from 2010 census; GHSL estimate for 2014; DoU estimate for 2015), Table S10:Three-way cross-classifications of official urban–rural categories, GHSL–official classification and DoU classes, India. Upper panel reflects Global DoU based on subdistricts and lower panels reflect DoU using settlement-level administrative data (official figures from 2010 census; GHSL estimate for 2014; DoU estimate for 2015), Figure S1: Degree of urbanization, New Delhi and surrounding areas. Left panel shows DoU distribution using the global data product based on sub-district-level population data; Right panel shows DoU distribution produced with the settlement-level population data, Figure S2: Alluvial Plots India, 25% and 1% [file NIHMS1770654-supplement-Harmonization_Supplementary.pdf]

remote sensing

MDPI

Supplement to "Global harmonization of urbanization measures: Proceed with care"

By Deborah Balk, Stefan Leyk, Mark R. Montgomery and Hasim Engin

This supplement illustrates ways in which the estimation is sensitivity to inputs, both the resolution of underlying inputs (in the administrative data) and in the choice of threshold of built-up levels. In the Appendix to the paper, we highlight areas of sensitivity, suggesting that of the officially urban locations, built-up levels of less than 50% may be quite reasonable. In this supplement, we consider two such lower levels, τ = 25% and 1% built-up.

Implications of using higher resolution administrative inputs for India

The analysis reported in the main body of the paper uses the global Degree of Urbanization (DoU) data product (Florczyk et al., 2019). For India, as population inputs, it uses publicly-available subdistrict-level administrative data (about 5,500 units). In this appendix, to illustrate how the spatial resolution of the underlying census data in the DoU impacts the resulting classification, the DoU method was applied[1] using the settlement level (about 650,000 units) boundaries and associated population described in the main text (Balk et al., 2019). The resulting spatial distribution can be seen in Figure S2 and Tables S1-S3.

It is well known that spatial resolution matters and gains in resolution tend to lead to more accurate estimates of population distributions (Tatem et al. 2011, Leyk et al, 2018, Leyk et al., 2019, Balk et al., 2009). In this illustration, because we also assume that the finer resolution data will produce a more accurate rendering of the spatial distribution of the population, we also assume that the resulting reallocation in the GHS-Pop data, which is an intermediary input to the DoU classification, will also produce a more accurate description of the population distribution of India. However, as we point out elsewhere (Balk et al., 2019) the spatial precision and accuracy of the Indian settlement-level boundaries is to some extent unknown. Data used here were matched with district and state borders, but we made the fewest alterations to the underlying boundaries as possible due to a comprehensive lack of objective criteria upon which to do so. It is worth noting that this is a more general concern globally: fine-resolution spatial data simply because they include orders of magnitudes more units provide ample opportunities for errors to go undetected. Small errors in fine resolution data could be amplified when integrated with other data that is also spatially refined (such as the built-up area data). So, while we expect qualitative improvements in resulting classifications, we expect some errors resulting from inconsistencies arising from combining multiple high-resolution spatial data.

Apart from the underlying data, in the application of the DoU method, Dijkstra et al. (2020) report that the size of the unit -- that is, the grid cell -- has an influence on the population density estimation used as part of the criteria to create the classification (see fn 15, on India vs. US), with coarser units resulting in lower average densities.

The left-panel map, for New Delhi and surrounding areas, is based on the coarser, subdistrict inputs and thus classifies more land (and corresponding population) as urban centers. It also renders more land as very sparsely or low-density rural. In contrast, on the right panel, based on settlement-level data with more detailed distributions of the popu-

[1] We thank our colleagues in the GHSL Team of the European Commission, DG Joint Research Centre – JRC E.1 Group, Disaster Risk Management Unit, for producing GHS-Pop and applying the DoU method to them.

lation, there is less reallocation underway. This produces more land area classified as suburban or peri-urban, as indicated clearly in the regions on the outskirts and expanding from this capital region.

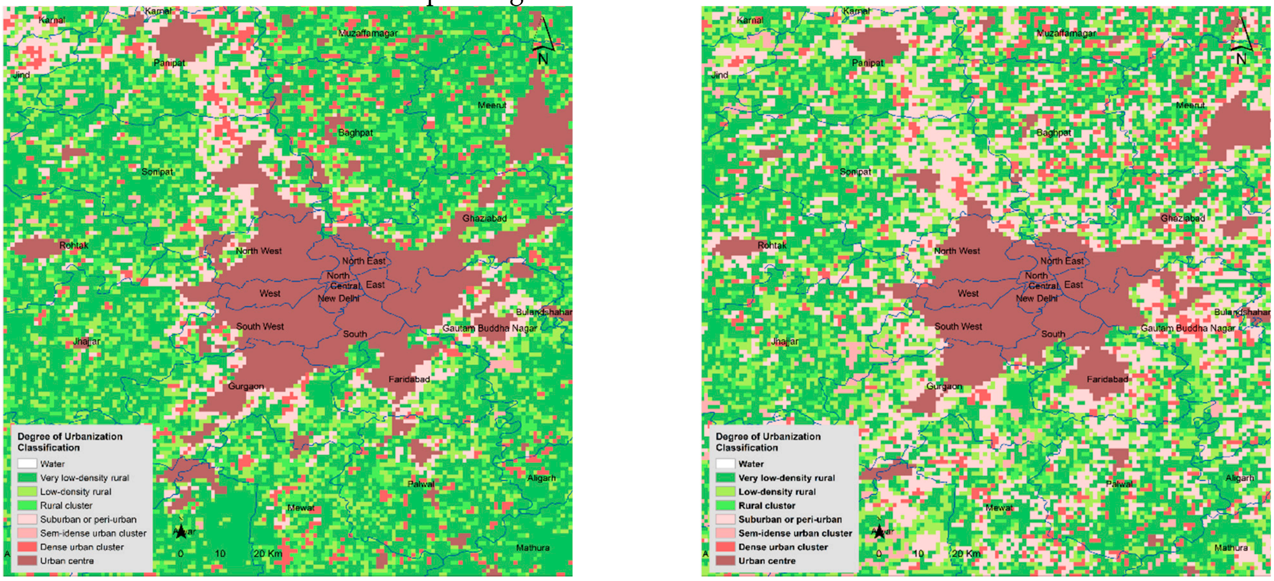

Figure S1. Degree of urbanization, New Delhi and surrounding areas (2015). Left panel shows DoU distribution using the global data product based on sub-district-level population data; Right panel shows DoU distribution produced with the settlement-level population data.

The pattern illustrated for New Delhi and surrounding areas is also reflected clearly in the national-level results, shown in Tables S1-S3. When using moderate resolution inputs, 2.3% of total land areas is estimated to be an urban center, in contrast to 1.6% when using the finer resolution inputs (Table S1). While these percentages are small, given that cities tend to occupy relatively small fractions of land in the first place, this difference is significant in relative terms. The estimated population living in urban centers, however, remains largely unchanged between the two approaches (Table S2).

Table S1. Total Area (km2) by Degree of Urbanization, using population data of varying spatial resolutions, India.

Degree of Urbanization Classification	Administrative-level used			
	Settlements		Subdistricts	
Urban centre	51,886	1.59%	75,094	2.31%
Dense urban	55,003	1.69%	51,522	1.58%
Semi-dense urban	54,909	1.68%	4,856	0.15%
Suburban or peri-urban	345,926	10.61%	41,477	1.27%
Rural	180,629	5.54%	234,587	7.21%
Low-density rural	821,116	25.19%	304,857	9.37%
Very low-density rural	1,750,424	53.70%	2,541,742	78.11%

One of most two notable differences is found in the suburban or peri-urban category, where the fine-resolution data estimate about 10 times more land area (Table S1) and 8 times more persons (Table S2) in that class. These very different urban forms, no doubt, would have significant implications for understanding urban life and future urbanization. The other notable difference that emerges is on the rural end of the continuum. The fine-resolution data estimate less land (Table S1) and population (Table S2) residing in all three rural classes, and perhaps most notably place 53.7% of India's land area and 23.5% of its population in "very low-density rural" areas. In contrast, the moderate-resolution inputs estimate 78.1% of India's land and 46.8% of its population live in these areas.

Table S2. Total Population (000) by Degree of Urbanization, using population data of varying spatial resolutions, India.

Degree of Urbanization Classification	Administrative-level used			
	Settlements		Subdistricts	
Urban centre	279,961	23.13%	296,221	24.52%
Dense urban	77,038	6.36%	45,516	3.77%
Semi-dense urban	25,168	2.08%	1,988	0.16%
Suburban or peri-urban	280,205	23.15%	38,501	3.19%
Rural	55,796	4.61%	128,965	10.68%
Low-density rural	207,360	17.13%	131,087	10.85%
Very low-density rural	284,868	23.54%	565,639	46.83%

Table S3 shows population densities, and leads to much different narratives. As the DoU based on the fine-resolution data reveals a smaller footprint for urban centers, the population density of such locations is even greater than estimated by moderate-level inputs. It is notably higher by more than 1000 persons/km2. Similarly, densities of 'dense urban' and 'semi-dense urban' areas are also higher when using the fine-resolution data. Conversely, suburban/peri-urban areas and all of the rural classes are less densely populated (despite much less land area having been estimated in these classes), though they still remain high compared to these classes for Mexico or the United States.

Table S3. Total Density (persons/km2) by Degree of Urbanization, using population data of varying spatial resolutions, India.

Degree of Urbanization Classification	Administrative-level used	
	Settlements	Subdistricts
Urban centre	5,396	3,945
Dense urban	1,401	883
Semi-dense urban	458	409
Suburban or peri-urban	810	928
Rural	309	550
Low-density rural	253	430
Very low-density rural	163	223

On one hand, the value-added by the remote sensing data inputs is perhaps greatest where census administrative inputs are coarse, because they help to create necessary spatial refinements. But on the other hand, integrated data models such as the DoU stand to be most accurate when built on a baseline of fine-resolution census inputs. (This also raises a related question of whether there is an optimal spatial resolution of census inputs in DoU models.) This illustration suggests that DoU classifications are dependent on the spatial resolution of the key input data, and while much attention has gone to the critical review of the satellite data underlying the DoU model, the spatial resolution of the census units clearly matters as well, and deserves critical and systematic review beyond this example here.

Sensitivity to built-up threshold (τ).

Tables 4-7 show the results of cross-classification based on inclusive built-up levels, using **τ** = 25 and 1 percent thresholds. As expected, more inclusive built-up levels lead to more land area (Table S4) and population (Table S5) for which there is urban agreement. Most notable is the case of India, for which 3 times the land area and more than twice the population falls into this category when going from a **τ** = 50% to a **τ** = 1%. Substantial increases are also observed in Mexico and the US, capturing another approximately 25%

of the total population as being cross-classified as urban agreement from around 57%. This suggests that flexible **τ** parameters would be useful in capturing officially urban areas along the continuum of rural-urban locations.

Table S4. Total (km2) and percentage area by cross-classifications of official urban designations with GHSL, using alternative built-up thresholds.

**Built-up threshold and agreement with census classification**		**Total Area (km²), Count and Percentage**					
		**India (2011)**		Mexico (2010)		USA (2010)	
**50% Built-up**	Urban agreement	12,580	0.4%	9,779	0.5%	113,853	1.5%
	Urban, not built-up	97,203	3.0%	13,164	0.7%	165,413	2.1%
	Rural, but built-up	5,014	0.2%	1,541	0.1%	13,618	0.2%
	Rural agreement	3,113,781	96.4 %	1,940,730	98.8%	7,517,111	96.2%
**25% Built-up**	Urban agreement	19,858	0.6%	13,237	0.7%	165,061	2.1%
	Urban, not built-up	89,926	2.8%	9,705	0.5%	114,205	1.5%
	Rural, but built-up	17,346	0.5%	4,872	0.2%	46,211	0.6%
	Rural agreement	3,101,448	96.1 %	1,937,399	98.6%	7,484,518	95.8%
**1% Built-up**	Urban agreement	40,767	1.3%	19,151	0.2%	240,984	3.1%
	Urban, not built-up	68,188	2.1%	3,791	1.0%	38,281	0.5%
	Rural, but built-up	140,077	4.3%	31,343	1.6%	341,479	4.4%
	Rural agreement	2,979,546	92.3 %	1,910,929	97.2%	7,189,250	92.1%

However, lower **τ** parameters also lead to more land and population classified as rural, but built-up reassigning them from areas of rural agreement. More research could help ascertain whether these areas are indeed rural in character (and how they could be described to be more accurately classified) or places that are the cusp of becoming reclassified as urban (Jones et al., 2020).

Table S5. Total (000s) and percentage population by cross-classifications of official urban designations with GHSL, using alternative built-up thresholds.

**Built-up threshold and agreement with census classification**		**Total Population (000) Count and Percentage**					
		**India (2011)**		**Mexico (2010)**		**USA (2010)**	
50% Built-up	Urban agreement	129,362	10.7%	60,550	57.0%	177,569	57.9%
	Urban, not built-up	247,747	20.5%	25,018	23.5%	69,948	22.8%

	Rural, but built-up	5,521	0.5%	96	0.1%	2,287	0.7%
	Rural agreement	828,225	68.4%	20,589	19.4%	56,854	18.5%
**25% Built-up**	Urban agreement	167,836	13.9%	70,638	66.5%	211,077	68.8%
	Urban, not built-up	209,273	17.3%	14,930	14.1%	36,439	11.9%
	Rural, but built-up	15,707	1.3%	278	0.3%	6,396	2.1%
	Rural agreement	818,039	67.6%	20,406	19.2%	52,745	17.2%
**1% Built-up**	Urban agreement	239,729	19.8%	81,718	76.9%	239,956	78.2%
	Urban, not built-up	134,741	11.1%	3,850	3.6%	7,561	2.5%
	Rural, but built-up	85,833	7.1%	1,429	1.3%	18,812	6.1%
	Rural agreement	750,551	62.0%	19,256	18.1%	40,329	13.2%

Of course, population (Table S6) and built-up (Table S7) densities are also responsive to alternative specifications of **τ**. Declines in all cross-classifications are evident as **τ** is lowered. Most notable is that while areas of Urban Agreement are pretty similar in all three study countries, once **τ** is lowered, India looks much different from Mexico and the US. At a built-up threshold of 1% areas of Urban Agreement in India are only 35% built-up as opposed to close to 50% in Mexico and the US. Whether this reflects differences in urban form, levels of economic development or satellite detection across the three countries remains an open question.

Table S6. Population density by cross-classifications of official urban designations with GHSL, using alternative built-up thresholds.

Built-up threshold and agreement with census classification		Persons/km²		
		India (2011)	Mexico (2010)	USA (2010)
**50% Built-up**	Urban agreement	10,283	6,192	1,560
	Urban, not built-up	2,549	1,901	423
	Rural, but built-up	1,101	62	168
	Rural agreement	266	11	8
**25% Built-up**	Urban agreement	8,452	5,336	1,279
	Urban, not built-up	2,327	1,538	319
	Rural, but built-up	905	57	138
	Rural agreement	264	11	7
**1% Built-up**	Urban agreement	5,881	4,267	996
	Urban, not built-up	1,976	1,016	198
	Rural, but built-up	613	46	55

Rural agreement	252	10	6

Table S7. Built-up density by cross-classifications of official urban designations with GHSL, using alternative built-up thresholds.

**Built-up threshold and agreement with census classification**		**Mean Built-up %**		
		**India (2011)**	**Mexico (2010)**	**USA (2010)**
**50% Built-up**	Urban agreement	78.3	82.0	79.5
	Urban, not built-up	4.7	14.4	16.7
	Rural, but built-up	66.8	66.5	67.5
	Rural agreement	0.4	0.2	0.4
**25% Built-up**	Urban agreement	63.0	70.27	66.4
	Urban, not built-up	2.1	6.5	7.5
	Rural, but built-up	44.0	44.8	44.3
	Rural agreement	0.3	0.1	0.6
**1% Built-up**	Urban agreement	35.3	51.8	49.0
	Urban, not built-up	0.0	0.1	0.1
	Rural, but built-up	11.3	12.7	12.1
	Rural agreement	0.0	0.0	0.0

Alluvial Plots: Sensitivity to alternative built-up thresholds, and to input-resolution of India census data.

The sensitivity to built-up threshold can also be seen in Figures S2 for India, S3 for Mexico and S4 for the U.S. showing **τ**= 25% and 1%, respectively. It is clear that as **τ** is low, allowing for more inclusive criteria for considering built-up land as likely urban, we see many more officially *rural areas that are built-up*. In India, this class according to DoU is heterogeneous, but in the U.S., it is largely classified as low-density rural, suggesting that 1% built-up may have a more urban signature in some settings than in others. Similarly, the class of *urban, not built-up* declines as **τ** decreases. In all countries, this is heterogeneous class: when it diminishes, it places those persons into the *urban centre* class, which owing to its dominance of population for whom there is *urban agreement*, it is hard to disentangle the contribution of the alternative **τ** levels to that class.

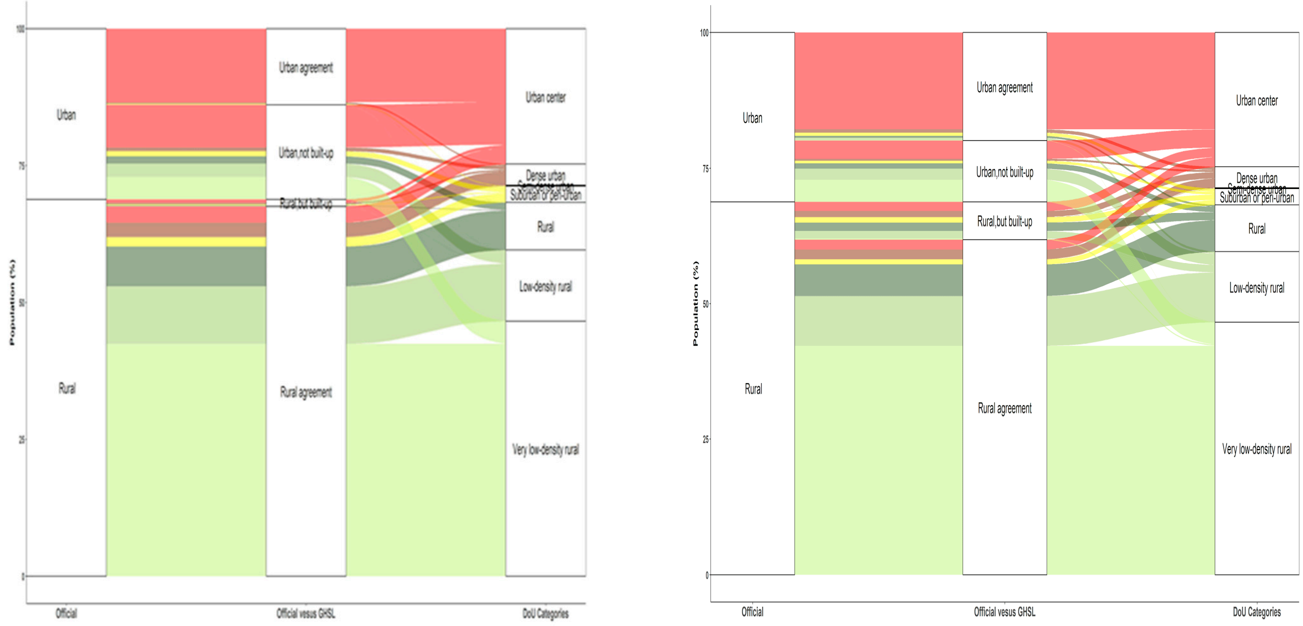

Figure S2. Alluvial Plots India, 25% and 1% thresholds.

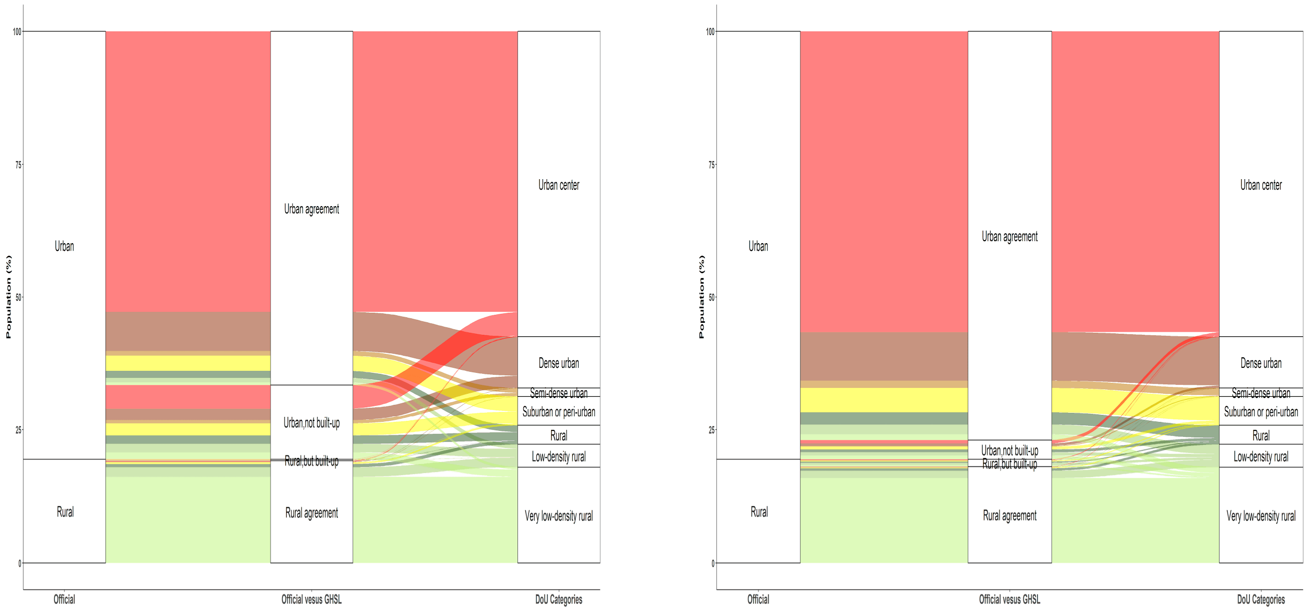

Figure S3. Alluvial Plots Mexico, 25% and 1% thresholds.

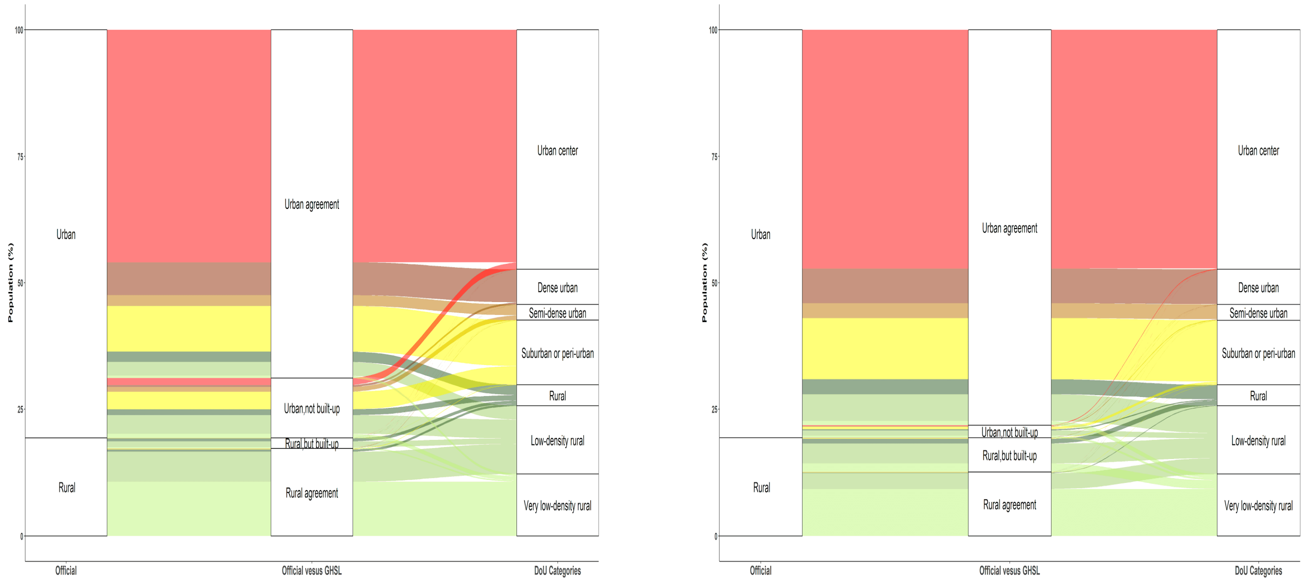

Figure S4. Alluvial Plots USA, 25% and 1% thresholds.

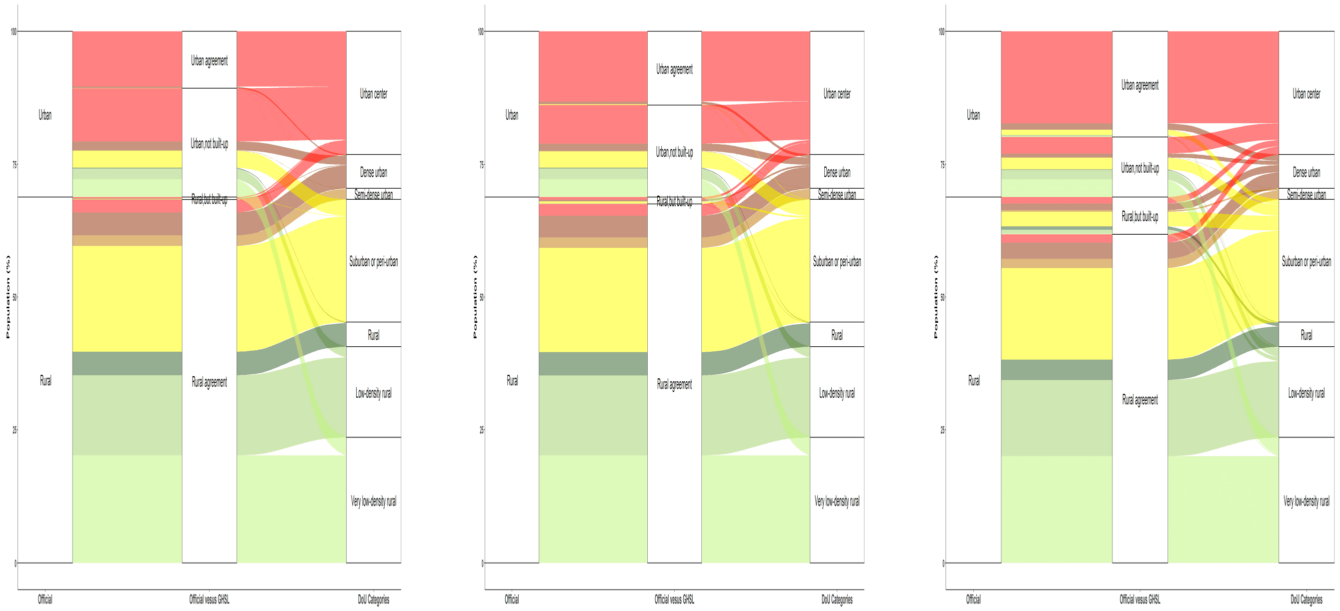

Figure S5. Alluvial Plots India using settlement-level population data, **τ** = 50% 25% and 1% thresholds, respectively.

Data corresponding to Figure 7 in the main text are shown below in Table S8-S10 below. Note that Table S10 also includes the finer resolution data for India, as part of an additional sensitivity analysis.

Table S8. Three-way cross-classifications of official urban–rural categories, GHSL–official classification, and DoU classes, Mexico. (Official figures from 2010 census; GHSL estimate for 2014; DoU estimate for 2015.).

Rural - Urban Classification Schema			Population		Area	
Official	Official vs. GHSL (**τ**=50%)	Degree of Urbanization	Count	%	Km²	%
Urban		Urban center	50,570,955	47.7	6,900	0.35

	Urban agreement	Dense urban	5,918,316	5.6	1,379	0.07
		Semi-dense urban	589,437	0.6	225	0.01
		Suburban or peri-urban	1,814,486	1.7	602	0.03
		Rural	764,570	0.7	334	0.02
		Low-density rural	498,285	0.5	192	0.01
		Very low-density rural	268,959	0.3	104	0.01
	Urban, not built-up	Urban center	10,069,152	9.5	3,076	0.16
		Dense urban	4,231,275	4.0	1,627	0.08
		Semi-dense urban	1,012,945	1.0	621	0.03
		Suburban or peri-urban	3,599,607	3.4	2,309	0.12
		Rural	2,306,478	2.2	1,624	0.08
		Low-density rural	2,081,867	2.0	2,011	0.10
		Very low-density rural	1,637,906	1.5	1,833	0.09
Rural	Rural, but built-up	Urban center	27,181	0.0	202	0.01
		Dense urban	4,088	0.0	74	0.00
		Semi-dense urban	2,593	0.0	55	0.00
		Suburban or peri-urban	20,631	0.0	249	0.01
		Rural	20,401	0.0	444	0.02
		Low-density rural	15,303	0.0	342	0.02
		Very low-density rural	4,846	0.0	162	0.01
	Rural agreement	Urban center	193,817	0.2	2,047	0.11
		Dense urban	80,893	0.1	1,660	0.09
		Semi-dense urban	74,287	0.1	1,637	0.08
		Suburban or peri-urban	383,091	0.4	5,849	0.30
		Rural	693,452	0.7	16,861	0.87

		Low-density rural	1,933,475	1.8	50,320	2.59
		Very low-density rural	17,113,828	16.2	1,842,629	94.72

Table S9. Three-way cross-classifications of official urban–rural categories, GHSL–official classification, and DoU classes, USA. (Official figures from 2010 census; GHSL estimate for 2014; DoU estimate for 2015.).

**Rural - Urban Classification Schema**			**Population**		**Area**	
Official	Official vs. GHSL **τ**=50%)	Degree of Urbanization	Count	%	Km²	%
Urban	Urban agreement	Urban center	130,623,637	42.8	67,091	0.87
		Dense urban	16,906,636	5.5	12,740	0.16
		Semi-dense urban	3,960,090	1.3	3,806	0.05
		Suburban or peri-urban	16,355,634	5.4	15,439	0.20
		Rural	3,759,080	1.2	4,066	0.05
		Low-density rural	4,025,917	1.3	7,282	0.09
		Very low-density rural	763,456	0.3	2,635	0.03
	Urban, not built-up	Urban center	13,776,616	4.5	22,040	0.29
		Dense urban	4,063,286	1.3	5,505	0.07
		Semi-dense urban	5,350,175	1.8	8,993	0.12
		Suburban or peri-urban	21,950,368	7.2	39,849	0.52
		Rural	5,901,485	1.9	11,903	0.15
		Low-density rural	15,508,722	5.1	56,759	0.73
		Very low-density rural	3,052,633	1.0	19,331	0.25
Rural	Rural, but built-up	Urban center	16,355	0.0	385	0.00
		Dense urban	22,352	0.0	275	0.00
		Semi-dense urban	22,569	0.0	212	0.00
		Suburban or peri-urban	49,851	0.0	599	0.01

		Rural	1,110,515	0.4	1,752	0.02
		Low-density rural	1,298,186	0.4	5,222	0.07
		Very low-density rural	265,278	0.1	5,019	0.06
	Rural agreement	Urban center	40,879	0.0	1,491	0.02
		Dense urban	45,644	0.0	976	0.01
		Semi-dense urban	212,412	0.1	3,361	0.04
		Suburban or peri-urban	445,248	0.1	8,146	0.11
		Rural	2,133,724	0.7	13,701	0.18
		Low-density rural	20,114,613	6.6	334,946	4.33
		Very low-density rural	33,166,925	10.9	7,076,286	91.55

Table S10. Three-way cross-classifications of official urban–rural categories, GHSL–official classification, and DoU classes, India. Upper panel reflects Global DoU based on subdistricts, and lower panels reflect DoU using settlement-level administrative data. (Official figures from 2010 census; GHSL estimate for 2014; DoU estimate for 2015.).

	**Rural - Urban Classification Schema**			**Population**		**Area**	
	**Official**	**Official vs. GHSL (τ=50%)**	**Degree of Urbanization**	**Count**	**%**	**Km²**	**%**
**India (based on sub-district level administrative data for DoU)**	Urban	Urban agreement	Urban center	126,969,757	10.5	11,707	0.36
			Dense urban	1,703,605	0.1	657	0.02
			Semi-dense urban	13,975	0.0	6	0.00
			Suburban or peri-urban	544,317	0.0	151	0.00
			Rural	52,913	0.0	22	0.00
			Very low-density rural	10,777	0.0	14	0.00
			Low-density rural	61,780	0.0	21	0.00
		Urban, not built-up	Urban center	129,225,897	10.7	28,416	0.88
			Dense urban	9,977,351	0.8	5,291	0.16
			Semi-dense urban	177,541	0.0	118	0.00

			Suburban or peri-urban	12,429,389	1.0	5,137	0.16
			Rural	16,015,567	1.3	7,765	0.24
			Very low-density rural	49,809,393	4.1	34,978	1.09
			Low-density rural	29,506,245	2.4	15,095	0.47
	Rural	Rural, but built-up	Urban center	3,137,590	0.3	1,941	0.06
			Dense urban	1,390,631	0.1	1,485	0.05
			Semi-dense urban	58,610	0.0	138	0.00
			Suburban or peri-urban	543,592	0.0	610	0.02
			Rural	269,948	0.0	642	0.02
			Very low-density rural	11,680	0.0	18	0.00
			Low-density rural	61,913	0.0	107	0.00
		Rural agreement	Urban center	39,403,869	3.3	32,888	1.02
			Dense urban	33,518,468	2.8	44,242	1.37
			Semi-dense urban	1,869,384	0.2	4,371	0.14
			Suburban or peri-urban	22,436,401	1.9	31,044	0.96
			Rural	88,673,377	7.3	182,591	5.67
			Very low-density rural	512,760,264	42.5	2,474,191	76.85
			Low-density rural	127,232,327	10.5	335,695	10.43
**India (based on settlement level administrative data for**	Urban	Urban agreement	Urban center	125,655,272	10.4	10,984	0.34
			Dense urban	3,280,001	0.3	1,314	0.04
			Semi-dense urban	12,184	0.0	18	0.00
			Suburban or peri-urban	381,337	0.0	241	0.01
			Rural	6,485	0.0	7	0.00
			Low-density rural	23,011	0.0	16	0.00

		Very low-density rural	3,605	0.0	2	0.00
	Urban, not built-up	Urban center	121,030,785	10.0	22,295	0.69
		Dense urban	20,341,163	1.7	8,843	0.27
		Semi-dense urban	731,300	0.1	827	0.03
		Suburban or peri-urban	38,378,160	3.2	17,922	0.56
		Rural	2,010,488	0.2	1,618	0.05
		Low-density rural	24,248,291	2.0	14,435	0.45
		Very low-density rural	40,815,460	3.4	31,112	0.96
Rural	Rural, but built-up	Urban center	2,689,663	0.2	1,331	0.04
		Dense urban	1,455,581	0.1	1,252	0.04
		Semi-dense urban	120,052	0.0	262	0.01
		Suburban or peri-urban	1,002,078	0.1	1,286	0.04
		Rural	191,295	0.0	606	0.02
		Low-density rural	54,952	0.0	205	0.01
		Very low-density rural	7,660	0.0	72	0.00
	Rural agreement	Urban center	30,585,257	2.5	17,198	0.53
		Dense urban	51,961,256	4.3	43,538	1.35
		Semi-dense urban	24,303,967	2.0	53,734	1.67
		Suburban or peri-urban	240,443,117	19.9	326,196	10.12
		Rural	53,587,378	4.4	178,201	5.53
		Low-density rural	183,033,742	15.1	805,075	24.97
		Very low-density rural	244,040,910	20.2	1,685,950	52.29

References

Balk, D.; Montgomery, M.R.; Engin, H.; Lin, N.; Major, E.; Jones, B. Urbanization in India: Population and Urban Classification Grids for 2011. *Data* 2019, 4. https://doi.org/10.3390/data4010035.

Balk, D. M. R. Montgomery, G. McGranahan, and M. Todd, 2009. "Understanding the Impacts of Climate Change: Linking Satellite and Other Spatial Data with Population Data," In G. Martine, J.M. Guzman, G. McGranahan, D. Schensul, and C. Tacoli (editors), *Population Dynamics and Climate Change*, New York: United Nations Population Fund and International Institute for the Environment and Development, pp. 206-217. http://www.environmentportal.in/files/pop_dynamics_climate_change.pdf#page=217

Dijkstra, L.; Florczyk, A.J.; Freire, S.; Kemper, T.; Melchiorri, M.; Pesaresi, M.; Schiavina, M. Applying the Degree of Urbanisation to the globe: A new harmonised definition reveals a different picture of global urbanisation. *Journal of Urban Economics* 2020, p. 103312. doi:https://doi.org/10.1016/j.jue.2020.103312.

Florczyk, A.; Corbane, C.; Ehrlich, D.; Freire, S.; Kemper, T.; Maffenini, L.; Melchiorri, M.; Pesaresi, M.; Politis, P.; Schiavina, M.; Sabo, F.; Zanchetta, L. GHSL Data Package 2019. Technical report, EUR 29788EN, Publications Office of the European Union, Luxembourg, 2019. doi:doi:10.2760/062975.

Jones, B.; Balk, D.; Leyk, S. 2020. Urban Change in the United States, 1990–2010: A Spatial Assessment of Administrative Reclassification. *Sustainability*, 12, 1–20. https://doi.org/10.3390/su12041649

Leyk, S., Gaughan, A. E., Adamo, S. B., de Sherbinin, A., Balk, D., Freire, S., Rose, A., Stevens, F. R., Blankespoor, B., Frye, C., Comenetz, J., Sorichetta, A., MacManus, K., Pistolesi, L., Levy, M., Tatem, A. J., and Pesaresi, M.: The spatial allocation of population: a review of large-scale gridded population data products and their fitness for use, *Earth Syst. Sci. Data*, 11, 1385–1409, https://doi.org/10.5194/essd-11-1385-2019, 2019.

Tatem AJ, Campiz N, Gething PW, Snow RW, Linard C. The effects of spatial population dataset choice on estimates of population at risk of disease. *Popul Health Metr*. 2011 Feb 7;9:4. doi: 10.1186/1478-7954-9-4.

List of Supplemental Tables & Figures

Table S1. Total Area (km2) by Degree of Urbanization, using population data of varying spatial resolutions, India.

Table S3. Total Density (persons/km2) by Degree of Urbanization, using population data of varying spatial resolutions, India.

Table S4. Total (km2) and percentage area by cross-classifications of official urban designations with GHSL, using alternative built-up.

Table S5. Total (000s) and percentage population by cross-classifications of official urban designations with GHSL, using alternative built-up thresholds.

Table S6. Population density by cross-classifications of official urban designations with GHSL, using alternative built-up thresholds.

Table S7. Built-up density by cross-classifications of official urban designations with GHSL, using alternative built-up thresholds.

Table S8. Three-way cross-classifications of official urban–rural categories, GHSL–official classification, and DoU classes, Mexico. (Official figures from 2010 census; GHSL estimate for 2014; DoU estimate for 2015.)

Table S9. Three-way cross-classifications of official urban–rural categories, GHSL–official classification, and DoU classes, USA. (Official figures from 2010 census; GHSL estimate for 2014; DoU estimate for 2015.)

Table S10. Three-way cross-classifications of official urban–rural categories, GHSL–official classification, and DoU classes, India. Upper panel reflects Global DoU based on subdistricts, and lower panels reflect DoU using settlement-level administrative data. (Official figures from 2010 census; GHSL estimate for 2014; DoU estimate for 2015.)

Figure S1. Degree of urbanization, New Delhi and surrounding areas. Left panel shows DoU distribution using the global data product based on sub-district-level population data; Right panel shows DoU distribution produced with the settlement-level population data.

Figure S2. Alluvial Plots India, 25% and 1% thresholds.

Figure S3. Alluvial Plots Mexico, 25% and 1% thresholds.

Figure S4. Alluvial Plots USA, 25% and 1% thresholds.

Figure S5. Alluvial Plots India using settlement-level population data, $\boldsymbol{\tau}$ = 50% 25% and 1% thresholds, respectively.
